# Lipopolysaccharide- TLR-4 Axis regulates Osteoclastogenesis independent of RANKL/RANK signaling

**DOI:** 10.1186/s12865-021-00409-9

**Published:** 2021-03-25

**Authors:** Mohammed S. AlQranei, Linda T. Senbanjo, Hanan Aljohani, Therwa Hamza, Meenakshi A. Chellaiah

**Affiliations:** 1 Department of Oncology and Diagnostic Sciences, School of Dentistry, University of Maryland, 650 W Baltimore Street, Baltimore, MD 21201 USA; 2 Preventive Dental Sciences Department, School of Dentistry, Imam Abdulrahman Bin Faisal University, Dammam, Saudi Arabia; 3 Department of Oral Medicine and Diagnostics Sciences, King Saud University, School of Dentistry, Riyadh, Kingdom of Saudi Arabia

**Keywords:** RANK ligand, Osteoclasts, Lipopolysaccharides, Bone Resorption

## Abstract

**Background:**

Lipopolysaccharide (LPS) is an endotoxin and a vital component of gram-negative bacteria’s outer membrane. During gram-negative bacterial sepsis, LPS regulates osteoclast differentiation and activity, in addition to increasing inflammation. This study aimed to investigate how LPS regulates osteoclast differentiation of RAW 264.7 cells in vitro.

**Results:**

Herein, we revealed that RAW cells failed to differentiate into mature osteoclasts in vitro in the presence of LPS. However, differentiation occurred in cells primed with receptor activator of nuclear factor-kappa-Β ligand (RANKL) for 24 h and then treated with LPS for 48 h (henceforth, denoted as LPS-treated cells). In cells treated with either RANKL or LPS, an increase in membrane levels of toll-like receptor 4 (TLR4) receptor was observed. Mechanistically, an inhibitor of TLR4 (TAK-242) reduced the number of osteoclasts as well as the secretion of tumor necrosis factor (TNF)-α in LPS-treated cells. RANKL-induced RAW cells secreted a very basal level TNF-α. TAK-242 did not affect RANKL-induced osteoclastogenesis. Increased osteoclast differentiation in LPS-treated osteoclasts was not associated with the RANKL/RANK/OPG axis but connected with the LPS/TLR4/TNF-α tumor necrosis factor receptor (TNFR)-2 axis. We postulate that this is because TAK-242 and a TNF-α antibody suppress osteoclast differentiation. Furthermore, an antibody against TNF-α reduced membrane levels of TNFR-2. Secreted TNF-α appears to function as an autocrine/ paracrine factor in the induction of osteoclastogenesis independent of RANKL.

**Conclusion:**

TNF-α secreted via LPS/TLR4 signaling regulates osteoclastogenesis in macrophages primed with RANKL and then treated with LPS. Our findings suggest that TLR4/TNF-α might be a potential target to suppress bone loss associated with inflammatory bone diseases, including periodontitis, rheumatoid arthritis, and osteoporosis.

**Supplementary Information:**

The online version contains supplementary material available at 10.1186/s12865-021-00409-9.

## Background

Inflammatory osteolytic lesions, including periodontitis and rheumatoid arthritis, impose severe health concerns. The primary outcome of osteolytic diseases is the loss of bone support [1, 2]. Inflammatory mediators’ infiltration into the inflamed region helps increase differentiation and osteoclast activity, thus increasing bone resorption [3–5].

Osteoclasts are multinucleated giant cells with bone resorption capabilities. These cells originate from mononuclear hematopoietic cells of the monocyte lineage [6]. Osteoclasts are highly motile cells that exhibit a unique functional cycle of migration, adhesion, and resorption [7]. Receptor activator of nuclear factor kappa-B ligand (RANKL) and macrophage colony-stimulating factor (M-CSF) are primary regulators of osteoclast differentiation [8, 9]. RANKL is highly expressed in osteoblasts [10]. Moreover, B cells, T cells, and fibroblasts of periodontal ligaments can secrete RANKL in response to inflammation [11–13]. Osteoprotegerin (OPG), a decoy receptor for RANKL, is secreted by osteoblasts, among other cells, and binds with RANKL with high affinity, thereby preventing the interaction of RANKL with its receptor RANK. Consequently, osteoclast differentiation and activation are blocked [8].

Bacterial lipopolysaccharide (LPS) is one of the most virulent factors triggering a local immune reaction [14]. It is a vital component of the outer membrane of gram-negative bacteria [15]. LPS is composed of three essential parts: 1) the outermost O- antigen, 2) the core oligosaccharides, and 3) the bioactive region, lipid A [16]. The interaction of LPS with mammalian cells stimulates the secretion of inflammatory cytokines, which subsequently leads to tissue destruction [17]. The primary cellular receptor that detects and interacts with LPS is toll-like receptor 4 (TLR4) [18].

TLRs are a class of receptors that regulate innate immune responses [19]. These receptors are characterized by their unique ability to detect various pathological molecular patterns, including lipoproteins, bacterial DNA, LPS, and double-stranded RNA. These receptors are commonly expressed in several cell types, including macrophages, dendritic cells, and neutrophils [20]. However, 10 human TLRs have been identified and named in a numerical sequence [21]. Among the TLRs, TLR4 is the primary receptor known to interact with LPS [18]. Pronounced secretion of pro-inflammatory mediators such as interleukin (IL)-6, tumor necrosis factor-α (TNF-α), and IL-1 is known to occur following LPS-TLR4 interaction [5, 22].

TNF-α is the dominant player in several inflammatory diseases, mediating innate and inflammatory responses [23]. It is secreted by macrophages and monocytes in response to inflammation and is implicated in many cellular events that lead to necrosis or apoptosis [24]. TNF-α induces its action upon binding to respective surface receptors, tumor necrosis factor receptor − 1 or − 2 (TNFR-1 or TNFR-2) [24]. TNF-α and RANKL are members of the TNF superfamily that share the same receptor family [25]. Furthermore, the signaling pathway involved with both proteins activates similar downstream targets such as mitogen-activated protein kinase (MAPK) and nuclear factor kappa B (NF-κB) [26]. Accumulating evidence suggests that TNF-α also plays a direct role in osteoclastogenesis [3, 27–30]. Moreover, numerous investigations have utilized LPS treatment to induce osteoclast differentiation [31–33]. However, the role of the LPS/TLR4/TNF-α axis in osteoclastogenesis is not comprehensively understood and warrants further elucidation.

In the present study, we revealed the potential role of LPS in forming osteoclasts from the RAW 264.7 (henceforth, denoted as RAW cells) murine macrophage cell line. LPS triggered osteoclast differentiation in RANKL-primed RAW cells by activating TLR4. However, treatment with LPS failed to induce osteoclastogenesis in a manner completely independent of RANKL. Additionally, the RANKL/RANK/OPG axis was not activated during LPS-mediated osteoclastogenesis. In RANKL-primed cells, the LPS-induced TNF-α secretion independently regulated osteoclast differentiation by possibly activating TNFR-2 but not TNFR-1. These data present a new inflammation-mediated mechanism of osteoclastogenesis that could be targeted to prevent excessive bone loss.

## Results

### LPS induces osteoclast differentiation in RANKL-primed RAW cells

Osteoclasts are multinucleated giant cells derived from the monocyte lineage [34]. RANKL is an essential mediator of osteoclast differentiation [9]. In the present study, we first evaluated the role of LPS in the induction of osteoclastogenesis independent of RANKL. We used the tartrate-resistant acid phosphatase (TRAP) enzyme staining method to detect terminally differentiated osteoclasts in all experiments. Undifferentiated RAW macrophage cells were also stained positive for TRAP because TRAP is expressed in mononuclear macrophage cells [35]. Accordingly, RAW cells treated with different doses of *Porphyromonas gingivalis* LPS (PG-LPS) (2, 5, and 10 μg/mL in medium) for 72 h were subjected to TRAP staining. RAW cells treated with RANKL alone were considered the control group (Additional file 1, Figure S1 A-C).

**Fig. 1:**
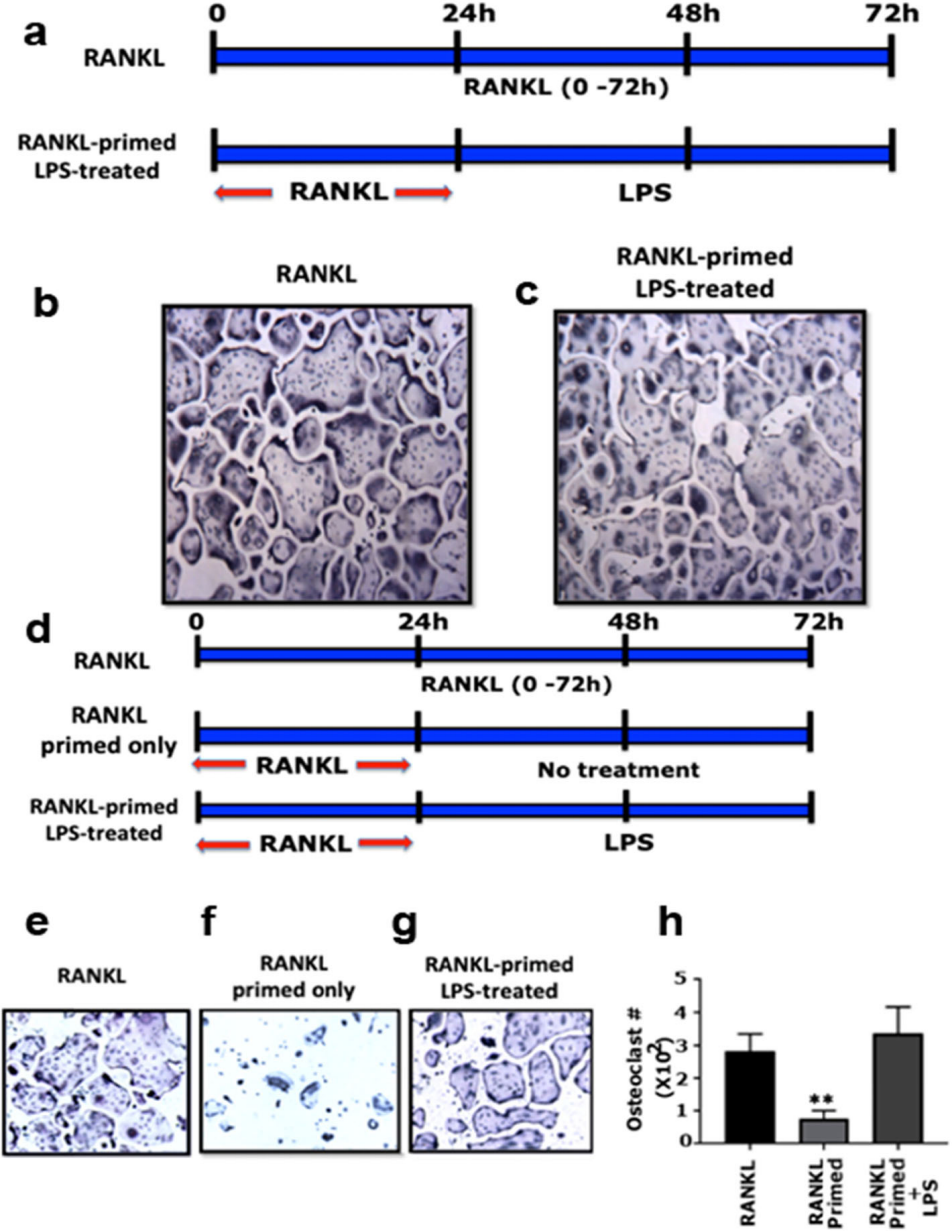
LPS induces osteoclast differentiation in RANKL-primed cells. **a** The diagrammatic sketch illustrates the treatment strategy related to RANKL and RANKL-primed LPS-treated groups. **b** and **c** Representative images of TRAP stained osteoclasts in response to the treatment strategy presented in panel A. **d** The diagrammatic sketch explains the treatment strategy performed to evaluate the osteoclastogenic ability of LPS. **e**, **f**, and **g** Representative images of TRAP stained osteoclasts in response to the treatment strategy presented in panel D. **h** The number of TRAP-positive multinucleated osteoclasts were counted in all treatment groups (*n* = 3). Statistical analysis was performed to compare the number of osteoclasts in the treatment groups with the control group (RANKL). One-way ANOVA was applied, and values are expressed as mean ± standard deviation (SD). **P < 0.01. Magnification is X100 in panels B, C, and E-G

Various in vitro studies have used different doses of PG-LPS, ranging from 2 μg/mL to 10 μg/mL, in their investigations [36–38]. Our results demonstrated that LPS alone failed to form osteoclasts even at higher doses (Additional file 1, Figure S1C). For efficient osteoclastogenesis, RAW cells need to be exposed to RANKL at 24 h intervals for 72 h. Therefore, we used different conditions in which RAW cells were treated with RANKL for 24 h (referred to as ‘primed with RANKL’) before adding LPS (5 μg/mL) for 48 h (Fig. 1a; schematic diagram). Compared with the addition of LPS alone, this experimental condition showed a dramatic increase in the number of osteoclasts, comparable with the RANKL control group (Fig. 1b and c). Therefore, for all results presented below with LPS, we followed the same PG-LPS treatment strategy at a 5 μg/mL concentration.

In the next set of experiments, we showed that primed RAW cells incubated with the serum-containing medium for an additional 48 h failed to differentiate into mature osteoclasts (Fig. 1f). Fully differentiated mature osteoclasts were observed in cultures treated with RANKL at 24 h intervals for 72 h (Fig. 1e) and in RANKL-primed cells treated with LPS for another 48 h (Panel G). Mature TRAP-positive osteoclasts were counted in approximately 4 to 5 fields/treatment from three different experiments and presented as a graph (*n* = 3) (Fig. 1h). The number of osteoclasts was significantly lower in cells primed with RANKL and incubated in the medium only for the next 48 h (Panels F and H). These results demonstrated the ability of LPS to promote osteoclast differentiation in RANKL-primed cells independent of RANKL. Notably, the RANKL-primed LPS-treated condition is henceforth denoted as *‘LPS-induced osteoclastogenesis.’*

### The RANK level is lower in LPS-induced osteoclasts as compared with RANKL-induced osteoclasts

**Fig. 2:**
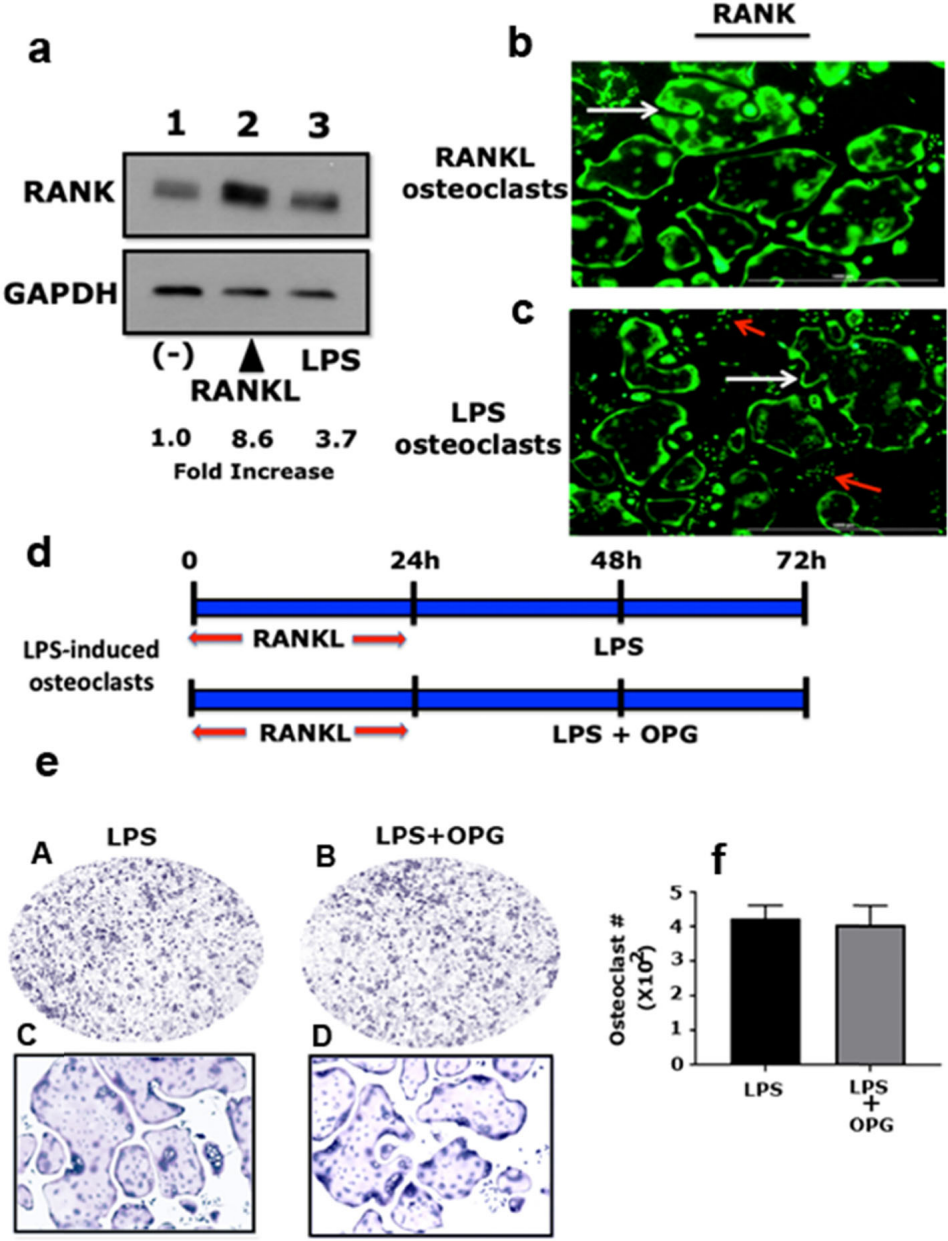
Analysis of RANK expression in LPS- and RANKL-mediated osteoclastogenesis. **a** Equal amounts of membrane lysate proteins were used for immunoblotting analyses with antibodies against RANK (**~** 90 kDa) and GAPDH (loading control; **~** 37 kDa). Protein levels were quantified by densitometry, corrected for the sample load based on the GAPDH level, and expressed as a fold increase relative to the control lane (−). The results represent one of three experiments performed. **b** and **c** Immunostaining with an antibody against RANK was performed in non-permeabilized RANKL (**b**) and LPS (**c**) -stimulated osteoclasts. White arrows indicate mature osteoclast. Red arrows indicate mononuclear cells. The results represent one of three experiments performed. **d** Identification of the time-dependent effect of OPG on LPS-induced osteoclast differentiation. The diagrammatic sketch demonstrates the treatment strategy of RAW cells with LPS (5 μg/mL) and OPG (120 ng/mL). **e** Representative images of TRAP stained osteoclasts in response to the treatment strategy is shown in panel D. TRAP stained osteoclasts in panels A and C were imaged with a 4× objective (magnification: 40X), and panels B and D were imaged with a 10× objective (magnification: 100X). **f** The number of TRAP-positive multinucleated osteoclasts were counted in both groups from three different experiments. Statistical analysis was performed to compare the number of osteoclasts in the LPS + OPG group with the control group (LPS). T-test was applied, and the difference between groups is not statistically significant. Scanned uncropped autoradiograms are presented in Additional file 5, Figure S5. Corresponding immunoblots are shown in panel A

RANK is a cell surface receptor for RANKL, and osteoclasts differentiate primarily via RANKL/RANK signaling [39]. Next, we sought to determine the RANK’s surface or membrane levels by immunoblotting and immunostaining analyses using an antibody against RANK (Fig. 2a and b). Membrane lysates were prepared to determine the surface or membrane levels of RANK. The immunoblotting study demonstrated that RANK levels were significantly higher in RANKL-induced osteoclasts (7.6-fold increase; Fig. 2a, lane 2) than in LPS-induced osteoclasts (2.7-fold increase; Fig. 2a, lane 3). An increase in RANK levels in LPS-induced osteoclasts was above the basal level observed in RAW cells (Fig. 2a; lane 1). We observed significantly fewer osteoclasts in RANKL-primed RAW cells (Fig. 1f). An increase in the number of osteoclasts in LPS-treated cells (Fig. 1g and h) indirectly suggests the influence of LPS on RANK expression (Fig. 2a, lane 3). The surface level of RANK was observed in the following order: RANKL>LPS> > RAW cells.

Immunostaining analyses with a RANK antibody corroborated the observations determined in the immunoblotting study (Fig. 2b and c). Figure 2c shows RANK staining in RAW cells (red arrows). The membrane distribution of RANK was higher in RANKL-induced osteoclasts (Fig. 2b, white arrow) than in LPS-induced osteoclasts (Fig. 2c, white arrow). The intensity of RANK was lower in membranes of osteoclasts induced by LPS than in RANKL-induced osteoclasts (Fig. 2c, white arrow). Further analyses are required to determine whether LPS plays a role in the surface expression of RANK.

### RANKL/RANK/OPG axis is not involved in LPS-induced osteoclastogenesis

OPG is an essential negative modulator of osteoclastogenesis. It binds to RANKL and thereby prevents its interaction with RANK [40]. To further confirm RANKL/RANK signaling’s passive role in LPS-induced osteoclasts, we added OPG to the culture conditions containing RANKL and LPS. A schematic diagram in (Additional file 2, Figure S2A) shows the treatment strategy of RAW cells with RANKL and OPG (120 ng/mL). Herein, we used two different conditions for OPG treatment in the presence of RANKL, termed as ‘late’ and ‘early + late’ (Additional file 2, Figure S2A). In these strategies, OPG was added for 48 h after the 24 h treatment with RANKL *(late)* or at the time of RANKL addition at 0 h *(early + late*). The incubation was continued for 72 h under both conditions, as described in the Material and Methods section. OPG was added at a 2:1 ratio with RANKL [41]. After 72 h of incubation, cells were stained for TRAP and analyzed. As expected, OPG attenuated RANKL-induced osteoclastogenesis (Additional file 2, Figure S2B) and reduced the number of TRAP-positive multinucleated osteoclasts in a time-dependent manner (Additional file 2, Figure S2C).

The next objective was to determine whether the membrane localization of RANK facilitates LPS-mediated osteoclastogenesis. We evaluated whether the addition of OPG and LPS at the same ratio would influence LPS-mediated osteoclastogenesis if RANK was involved. RAW cells were treated with OPG and LPS, as shown in the schematic representation (Fig. 2d). Intriguingly and unexpectedly, we observed a comparable number of osteoclasts in both groups tested with and without OPG in the presence of LPS (Fig. 2e and f). OPG failed to inhibit LPS-induced osteoclastogenesis (Fig. 2e). Quantitative analysis of osteoclast numbers from three different experiments corroborated this observation between the two groups (Fig. 2f).

In the immunoblotting analysis, OPG reduced the membrane levels of RANK (0.7-fold decrease) in the membrane fraction of LPS + OPG-treated cells (Additional file 3, Figure S3A, lanes 3 and 4); however, osteoclast differentiation was not affected (Fig. 2e and f). Nevertheless, a decrease in RANK levels in RANKL+OPG-treated cells (0.7-fold decrease; Additional file 3, Figure S3A, lanes 1 and 2) corresponded well with decreased osteoclastogenesis (Additional file 2, Figure S2B and C). These results demonstrate that LPS could induce osteoclast differentiation in RANKL-primed cells through different mechanisms, which may not require the LPS-mediated RANKL/RANK/OPG axis.

### Analysis of LPS mediated TLR4 activation on osteoclastogenesis

**Fig. 3:**
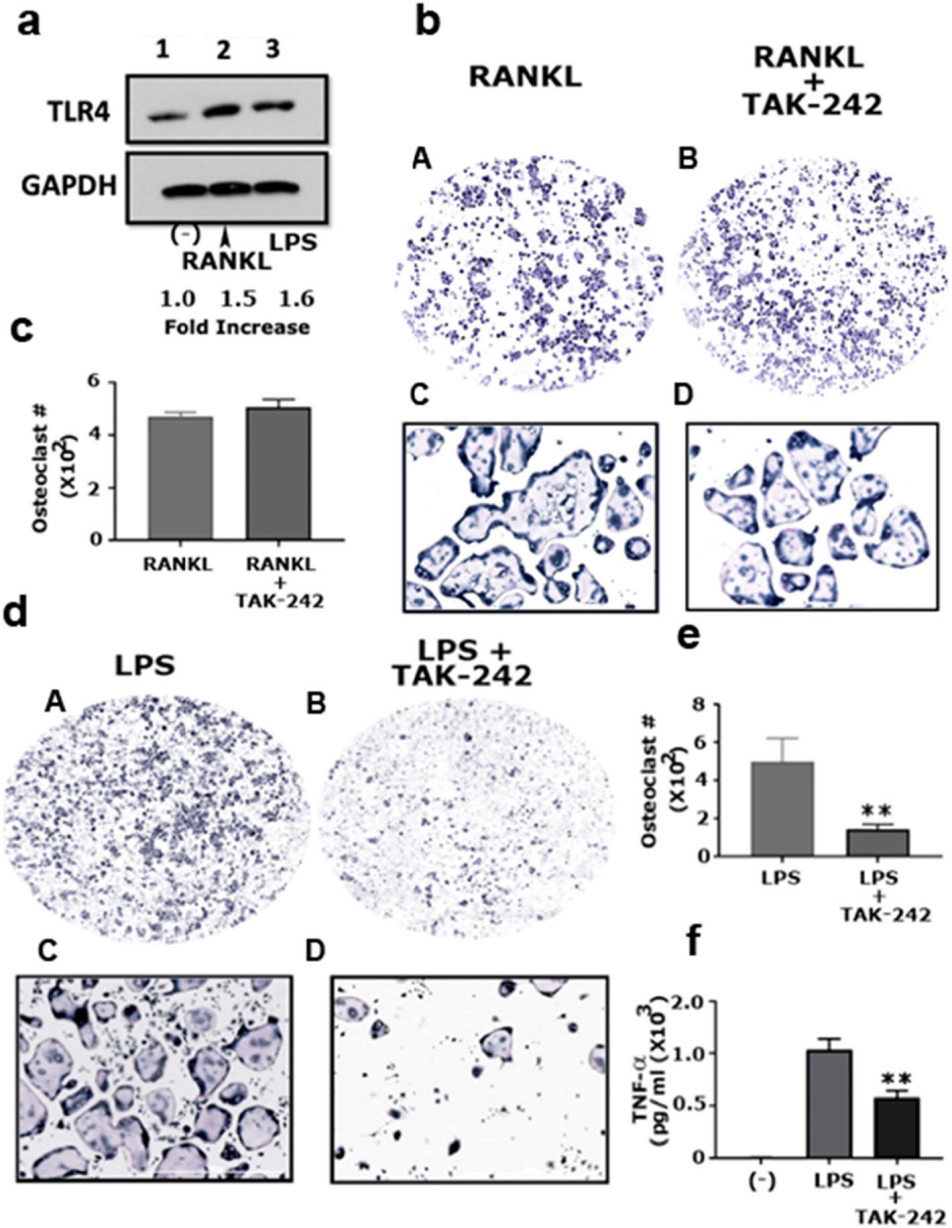
Evaluation of the involvement of TLR4 signaling in RANKL-induced osteoclastogenesis. **a** Immunoblotting analyses with antibodies against TLR4 (**~** 95 kDa) and GAPDH (loading control; **~** 37 kDa) are shown. Protein levels were quantified by densitometry, corrected for the sample load based on GAPDH expression, and expressed as a fold increase relative to the control lane (−). The results represent one of three experiments performed. **b** Representative images of TRAP stained osteoclasts in response to treatment with RANKL and TAK-242 (5 μM/mL). TRAP stained osteoclasts in panels A and B were obtained with a 4× objective (magnification: 40X), while those in panels C and D were obtained with a 10× objective (magnification: 100X). **c** The number of TRAP-positive multinucleated osteoclasts were counted in both groups (*n* = 3). Statistical analysis was performed to compare the number of osteoclasts in the RANKL+TAK-242 group with the control group (RANKL). The t-test was applied; the difference between groups is not statistically significant. **d** TRAP stained osteoclasts in response to treatment with LPS (5 μg/mL) and LPS/TAK-242 (5 μM/mL) are shown. TRAP stained osteoclasts in panels A and C were obtained with a 4× objective (magnification: 40X), while those in panels B and D were obtained with a 10× objective (magnification: 100X). **e** The number of TRAP-positive multinucleated osteoclasts were counted in both groups (*n* = 3). Statistical analysis was performed to compare the number of osteoclasts in the LPS + TAK-242 group with the control group (LPS) using the t-test. **P < 0.01 vs LPS group. **f** Effect of TAK-242 on LPS-induced TNF-α production from RAW cells-derived osteoclast. ELISA determined the concentrations of TNF-α in the culture medium. One-way ANOVA was applied, and values are expressed as mean ± standard deviation (SD). **P < 0.01 vs. LPS group. Scanned uncropped autoradiograms are presented in Additional file 5, Figure S5. Corresponding immunoblots are shown in panel A

Several reports have indicated the ability of the pro-inflammatory cytokine TNF-α to induce osteoclast differentiation by binding to its receptor [3, 27, 29]. Exposure of osteoclast precursors to bacterial endotoxins (e.g., LPS) may be one of the primary reasons for the secretion of TNF-α by these cells [20]. TLR4 is expressed on the surface of osteoclast precursors and is considered the primary receptor for LPS [18]. Therefore, we hypothesized that LPS binds to TLR4 in osteoclast precursors and causes the cells to secrete TNF-α, which mediates osteoclastogenesis in an autocrine/paracrine manner through TNFR signaling. To test this hypothesis, we first evaluated the membrane levels of TLR4 in RANKL- and LPS-induced osteoclasts by immunoblotting analysis. The surface level of TLR 4 remained approximately the same in both the RANKL and LPS groups (Fig. 3a, lanes 2 and 3). However, both groups showed relatively higher TLR4 expression (~ 0.5-fold increase) than the untreated RAW cells (Fig. 3a, lane 1), which indicates the possible involvement of TLR4 in osteoclastogenesis.

Next, we assessed whether interfering with TLR4 signaling could decrease LPS-mediated osteoclast formation. Therefore, we utilized TAK-242, a selective TLR4 inhibitor [41]. RAW cells were treated with TAK-242 (5 μM/mL) in the presence of RANKL or LPS for 48 h, as detailed in the Materials and Methods section. TAK-242 significantly reduced LPS-induced osteoclast formation (Fig. 3D, panels c, and d) compared with cells treated with LPS alone (Fig. 3D, panels a and b). However, TAK-242 did not affect RANKL-induced osteoclast formation (Fig. 3B, panels c and d). Quantitative analysis of osteoclast numbers from three different experiments is presented in Fig. 3c and e.

Previous investigators have revealed the osteoclastogenic function of the pro-inflammatory cytokine TNF-α. Therefore, to determine whether this inhibition can be attributed to the reduced secretion of LPS-mediated TNF-α, we analyzed TNF-α expression levels in the LPS and LPS + TAK-242 groups using enzyme-linked immunosorbent assay (ELISA) (Fig. 3f). The basal level of TNF-α was measured in unstimulated RAW cells (−). The control LPS group showed a dramatic increase in TNF-α levels (Fig. 3f), which corresponded with the increased number of osteoclasts (Fig. 3D, panels a and b). However, the addition of TAK-242 significantly attenuated the LPS-induced secretion of TNF-α (Fig. 3f), explaining the reduced number of osteoclasts observed in the same group (Fig. 3D, panels c and d). These results indicate a potential regulatory role of TLR4 in LPS-mediated osteoclastogenesis.

### TNF-α functions as an autocrine/paracrine factor in the regulation of osteoclastogenesis in LPS-stimulated RAW cells

**Fig. 4:**
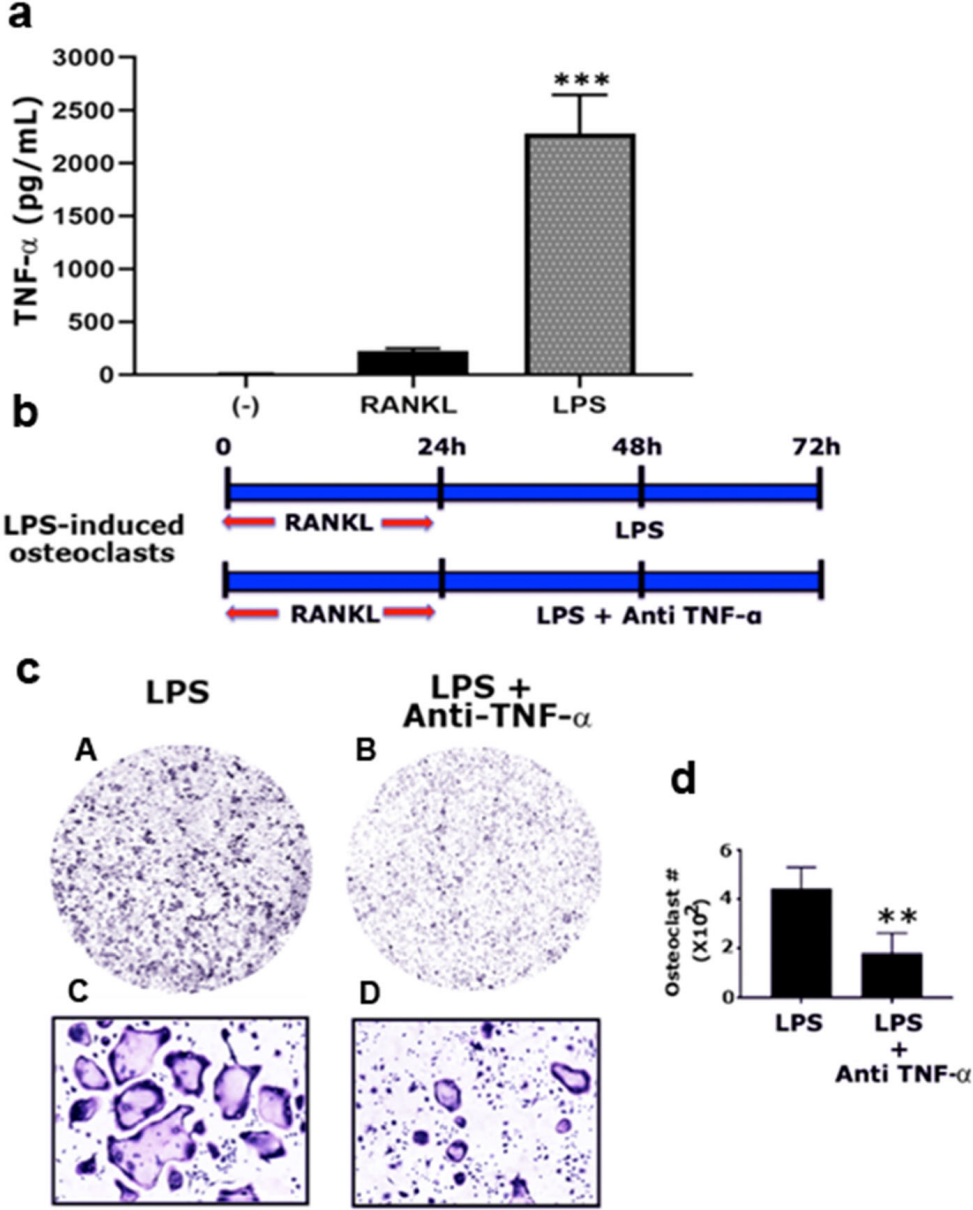
Analysis of the regulatory role of TNF-α in LPS- induced osteoclastogenesis. **a** Measurement of TNF-α production in response to RANKL and LPS stimulation of RAW cells. ELISA determined the TNF-α concentrations in the culture medium. One-way ANOVA was applied, and values are expressed as mean ± standard deviation (SD). ***P < 0.001 vs. (−) control and RANKL- treated cells. **b** The diagrammatic sketch demonstrates the treatment strategy of RAW cells with LPS (5 μg/mL) and anti-TNF-α (2 μg/mL). **c** In response to the treatment strategy, representative images of TRAP stained osteoclasts are shown in panel B. TRAP stained osteoclasts in panels A and C were obtained with a 4X objective (magnification: 40X); panels in B and D were obtained with a 10× objective (magnification: 100X). **d** The number of TRAP-positive multinucleated osteoclasts were counted in both groups (*n* = 3). Statistical analysis was performed to compare the number of osteoclasts in the LPS+ anti- TNF-α group with the control group (LPS). The t-test was applied, and values are expressed as mean ± standard deviation (SD). **P < 0.01 vs. LPS group. LPS, lipopolysaccharide; RANKL, receptor activator of nuclear factor kappa-B ligand; TNF-α, tumor necrosis factor α

Here, we performed ELISA to measure the levels of TNF-α secreted in cultures treated with RANKL and LPS. TNF-α was considerably more in cells treated with LPS than untreated (−) or RANKL-treated cells (Fig. 4a). To confirm the osteoclastogenic role of LPS-induced TNF-α, we treated RAW cells with a neutralizing antibody targeting TNF-α (2 μg/mL) in the presence of LPS in cells primed with RANKL for 24 h (Fig. 4b). A significant decrease in the number of mature osteoclasts was observed in osteoclasts treated with the neutralizing antibody targeting TNF-α cells (Fig. 4C - panels c and d) when compared with LPS-treated cells (Fig. 4C - panels a and b). Quantitative analysis of three different experiments is presented in the graph (Fig. 4d), which corroborated the results shown in Fig. 4c. Although anti-TNF- α significantly reduced osteoclast differentiation in the presence of TNF- α, it did not affect RANKL-induced osteoclastogenesis (Additional file 4, Figure S4). These results further confirmed the unique mechanism through which LPS regulates osteoclastogenesis by stimulating the secretion of TNF-α as an autocrine factor.

### TNF-α/TNFR-2 signaling regulates LPS-induced osteoclastogenesis

**Fig. 5:**
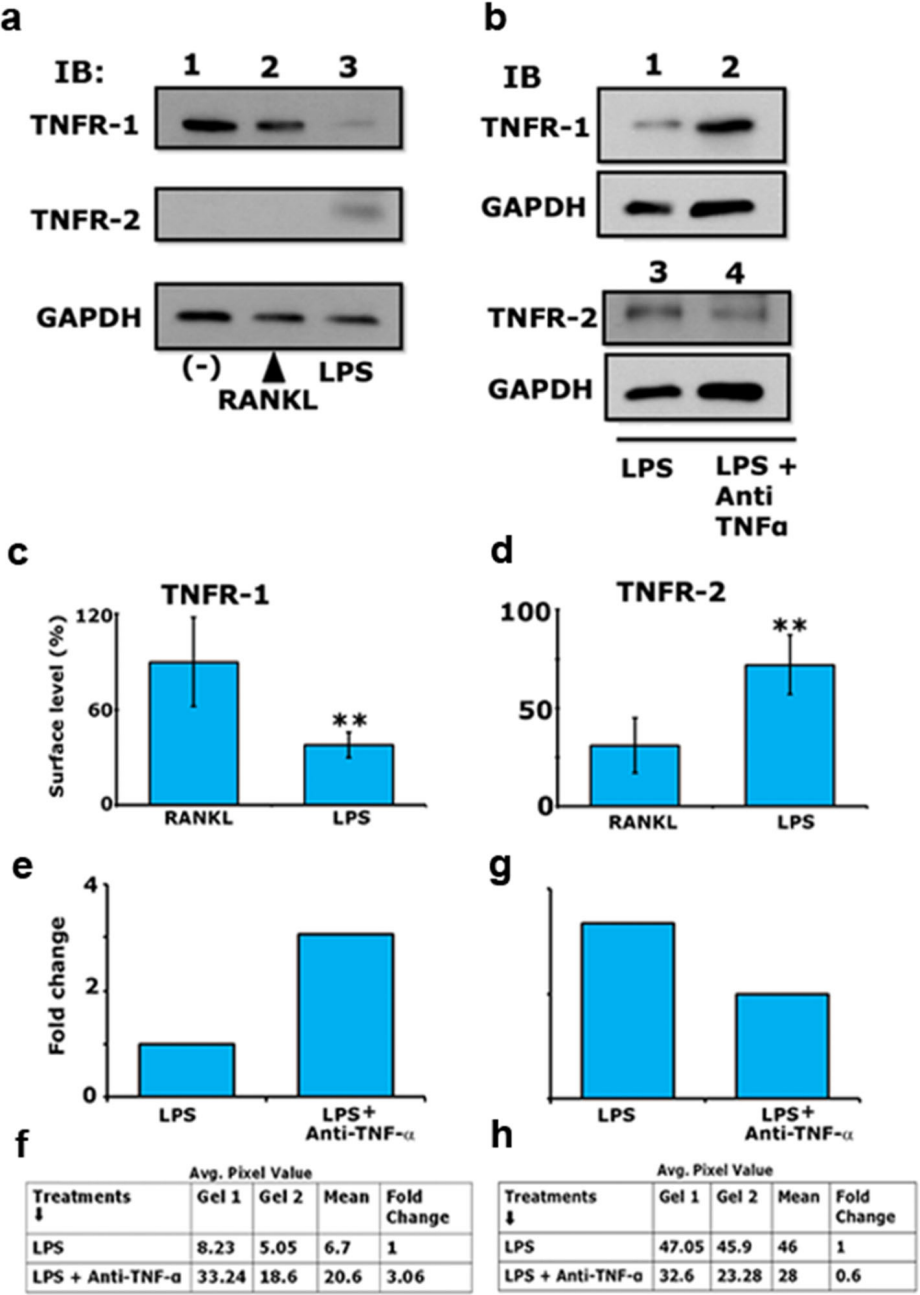
Effect of RANKL, LPS, and anti-TNF-α / LPS on the membrane levels of TNFR-1 and TNFR-2. Panels **a**, **c**, and **d** Immunoblotting analysis for TNFR1 (**a**; top panel, ~ 55 kDa) and TNFR-2 (middle panel in **a**, ~ 68 kDa) in osteoclasts differentiated with RANKL and LPS are shown. Representative immunoblotting showing the membrane levels of TNFR-1 and -2 (Panel **a**). Protein levels in untreated RAW cells (−) are shown in lane 1. Membrane levels of TNFR-1 (*n* = 4) and TNFR-2 (*n* = 3) were quantified in an Un-Scan IT software, corrected for the GAPDH level, and provided as percentage surface level of receptors (Panels **c** and **d**). **p < 0.001 vs. LPS-treated cells (**c** and **d**). One-way ANOVA was applied, and values are expressed as mean ± SD of four and three independent experiments for TNFR-1 and TNFR-2, respectively. TNFR-2 blot in A was stripped and blotted with a GAPDH antibody (bottom panel in **a**). Panels **b** and **e**-**h** Immunoblotting analysis demonstrates the effect of LPS (lane 1) and LPS+ neutralizing antibody to TNF-α (Lane 2) on the membrane levels of TNFR1 (**b**; top panel) and TNFR-2 (**b**; middle panel). Protein levels were quantified by densitometry, corrected for the sample load based on GAPDH level, and provided as a fold-change relative to LPS- treated control cells (Panels **e** and **g**). The Table (**f** and **h**) provides the average pixel value of the protein bands (TNFR-1 in **f** and TNFR-2 in **h**) from two experiments and fold changes in the surface levels of interest proteins. The experiment was performed twice and demonstrated remarkably related results. Raw data are provided in the (Additional file 7, Figure S7; A-C)

The functions of TNF-α are typically mediated via its receptors TNFR-1 or TNFR-2. TNFR-1 is the primary receptor that mediates a majority of TNF-α actions and is expressed in almost all cell types. Conversely, TNFR-2 has been less extensively evaluated, and its effect is mainly confined to some immune and tumor cells [42]. As an increase in the secretory levels of TNF-α was observed in LPS-induced osteoclasts (Fig. 4a) and a neutralizing antibody against TNF-α attenuated the effect mediated by LPS, we determined the receptor levels of TNF-α under the same conditions (Fig. 5a and b). Immunoblotting analysis of the membrane fraction was performed with an antibody against TNFR-1 or TNFR-2. Interestingly, the TNFR-1 level was more in RANKL-induced osteoclasts (Fig. 5a, lane 2) than in LPS-induced osteoclasts. About 60% decrease in the level of TNFR-1 was observed in osteoclasts differentiated with LPS (Fig. 5a, lane 3). However, a significant increase in the level of TNFR-2 was observed in LPS-induced osteoclasts (Fig. 5a, lane 3). The TNFR-2 protein was significantly lower in untreated or RANKL-induced cells (Fig. 5a, lanes 1 and 2).

Uncropped autoradiograms are provided in the (Additional file 7, Figure S7; A and B). Statistical analysis of the surface levels of TNFR-1 and TNFR-2 are shown in panels C and D.

Next, we determined the effect of anti-TNF-α on the surface levels of these receptors using immunoblotting analyses. We observed a decrease in the level of TNFR-2 in response to the LPS/anti-TNF-α treatment compared with LPS alone treated cells (Fig. 5b, lanes 3 and 4). In contrast, TNFR-1 levels were increased (Fig. 5b, lanes 1 and 2), which indicates a possible negative regulatory role of TNFR-1 in LPS-induced osteoclastogenesis. We observed a decrease in the levels of TNFR-2 in two experiments performed with a neutralizing antibody against TNF-α. Cumulative data from two experiments are provided as fold changes in the surface levels of TNFR-1 and TNFR-2 (Fig. 5e-h). Based on these results, we suggest that TNFR-2 may play a role in regulating LPS-induced osteoclastogenesis.

## Discussion

Bone loss is a significant outcome of inflammatory osteolytic lesions. The underlying mechanism is mostly related to the abnormal increase in osteoclast differentiation. During normal bone remodeling, a coupled and well-coordinated process between osteoblasts and osteoclasts sustains skeletal tissues’ health status. Some inflammatory disorders (e.g., periodontitis) cause bone loss owing to an imbalance in the activity of bone cells, mainly by increasing the differentiation and activity of osteoclasts [43]. Osteoporosis is a bone disease characterized by increased osteoclastic activity, resulting in decreased bone density [1]. Osteoclasts are typically differentiated following RANKL/RANK signaling [8]. In the present study, our data uncovered a unique mechanism that promotes osteoclast formation in vitro. We observed that TNF-α secreted via LPS/TLR4 signaling can induce osteoclast precursors to differentiate into fully mature osteoclasts in an autocrine/paracrine manner.

In this study, the osteoclast differentiation via RANKL and LPS was explored using the murine macrophage cell line, RAW 264.7. Notably, RANKL and M-CSF are primary regulators of osteoclastogenesis [9]. Herein, our key finding is that, although RANKL is the primary regulator of osteoclastogenesis, it was only required to shift the precursor cells to the osteoclast-like phenotype; then, LPS-induced TNF-α can independently sustain osteoclastogenesis. We believe that this could be one of the mechanisms that occur under conditions where inflammatory mediators (e.g., TNF-α) are secreted due to inflammation in the tissues of interest. These inflammatory mediators could mediate the differentiation of potent hematopoietic progenitors into osteoclasts, causing bone loss.

Lipopolysaccharides are vital characteristic components of the outer membrane or the cell wall of gram-negative bacteria [15]. During bacterial infections, LPS can invade tissues and interact with immune cells, causing the release of several pro-inflammatory mediators such as TNF-α and IL-1 [17]. The direct/indirect role of LPS in promoting osteoclastogenesis requires further elucidation. PG is one of the primary microorganisms responsible for periodontal inflammation [44]. Therefore, we used PG-derived LPS (PG-LPS) in our experiments to mimic inflammatory events occurring in periodontal infections.

RANK is part of the TNFR superfamily [45]. In the present study, we observed a significant decrease in the membrane expression of RANK in LPS-induced osteoclasts when compared with RANKL-treated cultures. OPG is a negative regulator of RANKL-induced osteoclastogenesis. It binds RANKL and thus suppresses its activity [40]. As anticipated, our data showed a potent effect of OPG in RANKL-treated cultures, resulting in osteoclastogenesis attenuation. Nonetheless, LPS continued to activate osteoclastogenesis despite the presence of OPG. Collectively, these data suggest the presence of a different mechanism for osteoclastogenesis other than the RANKL/RANK/OPG axis during LPS-mediated inflammatory events.

Several studies have demonstrated the dual role of LPS in osteoclastogenesis. For example, freshly isolated bone marrow macrophages (BMMs) failed to differentiate into osteoclasts after exposure to LPS [46]. However, priming the cells with RANKL followed by LPS stimulation resulted in a dramatic increase in osteoclast numbers. Unlike the RANKL priming effect, LPS primed cells could not undergo osteoclastogenesis after stimulation with RANKL [46]. Similarly, osteoclastogenesis inhibition was observed after culturing PG bacteria alone with BMMs derived from C57BL/6 mice. Additionally, when co-treated with RANKL, PG was able to block osteoclastogenesis. Pretreatment with RANKL was the only strategy that allowed PG to promote osteoclast differentiation in BMMs [47]. Consistent with these observations, our data showed that LPS alone failed to differentiate RAW cells into osteoclasts even at higher doses. Nevertheless, LPS promoted osteoclastogenesis in RAW cells treated with RANKL for 24 h (i.e., RANKL-primed cells).

In the present study, one objective was to investigate which receptors are activated during LPS-induced osteoclastogenesis. Therefore, to obtain further reliable data, we used membrane fraction lysates in all our immunoblotting analyses to determine the surface levels of receptors of interest. Although GAPDH is known as a cytosolic protein, it is also localized in the plasma membrane [48–50]. Thus, GAPDH was used as a loading control in our study. TLR4 is a surface receptor that activates the innate immune response. This TLR receptor can mediate bacterial LPS signaling [51]. Data on whether LPS binds TLR4, TLR2, or both remain inconclusive.

Nonetheless, many reports suggest that LPS exclusively acts through TLR4. The occurrence of TLR2 activation can be attributed to the presence of other bacterial contaminants, such as lipoproteins, in the isolated LPS from bacteria [37, 52, 53]. Therefore, in our experimental analysis, we used a specific TLR4 signaling inhibitor, TAK-242. This small-molecule inhibitor selectively binds to the intracellular domain of TLR4 and disrupts the interaction between TLR4 and its adapter molecules [41, 54]. During osteoclastogenesis, we observed a significant decrease in the secreted level of LPS-induced TNF-α following the treatment of RAW cells with TAK-242. This reduction was accompanied by reduced osteoclast differentiation. Our findings suggest that LPS acts primarily through TLR4 to regulate osteoclastogenesis.

Moreover, several studies have illustrated the differential roles of TLR4 and TLR2 in modulating osteoclastogenesis. For example, TLR2 activation by PG-LPS, but not TLR4, reportedly inhibits RANKL-induced osteoclast differentiation [47]. Furthermore, Takami et al. have demonstrated that TLR4 stimulation by LPS inhibits RANKL-induced osteoclastogenesis in RAW cells [55]. In contrast, one study has reported that activation of TLR4, but not TLR2, in response to inhaled organic dust, increased the osteoclast population and subsequent bone loss [56]. In addition to the role of TLR4 in modulating osteoclastogenesis, LPS activation of TLR4 enhances the survival of mature osteoclasts [57]. In the present study, we revealed a possible regulatory role of TLR4 in osteoclastogenesis. For the first time, we showed that in RANKL-primed cells, LPS activation of TLR4 substituted RANKL signaling triggered a parallel osteoclastogenic mechanism.

TNFR-1 and TNFR-2, as well as RANK, belong to the same receptor family. Generally, similar downstream targets are activated upon stimulation of the respective signaling mechanisms [26]. Therefore, TNFRs, especially TNFR-1, are often shown to regulate osteoclastogenesis [26, 29]. For instance, the regulation mediated by RANK and TNFR-1 coordinates a synergistic signaling mechanism in osteoclast formation. For example, common downstream signaling mediators TRAF6, TRAF2, and c-Src of TNF-α and RANKL are involved in enhancing osteoclast differentiation [26]. These downstream mediators were considerably reduced in cells transfected with TNFR-1. Additionally, activation of TNFR-1 by TNF-α dramatically increased osteoclast differentiation in RANKL-primed cells, whereas the deletion of TNFR-1 attenuated this process [26].

Studies have reported the differential roles of TNFRs in LPS-induced bone resorption. An in vivo study has revealed the decisive osteoclastogenic action of TNFR1 in response to an LPS injection, whereas TNFR-2 might have a preventive role against LPS-mediated bone loss [58]. Kobayashi et al. have demonstrated that TNFR-1 and TNFR-2 signaling is fundamental for TNF-α osteoclastogenesis. TNFR- knockout mice fail to generate osteoclasts after stimulation with TNF-alpha but not RANKL, indicating a possible TNF-α signaling role in RANKL-induced osteoclastogenesis [27]. LPS stimulates osteoclastogenesis via TNF-α with a potential part of TNFR1 [31]. Our findings suggest that LPS-induced TNF-α may signal through TNFR-2 to initiate osteoclastogenesis, while TNFR-1 seems to have a negative role in osteoclastogenesis. The limitations of this study are provided below. Future investigations must identify the affinity of TNF-α secreted by mature osteoclasts to TNFR1 or TNFR2. The specific roles of TNFR1 and TNFR2 have not been elucidated to come to a firm conclusion on the part of TNFR2 on LPS mediated osteoclastogenesis. Therefore, future experiments will use SiRNA and neutralizing antibody strategies to target TNFR1 or TNFR2 to validate their role on LPS-induced osteoclastogenesis.

## Conclusion

**Fig. 6:**
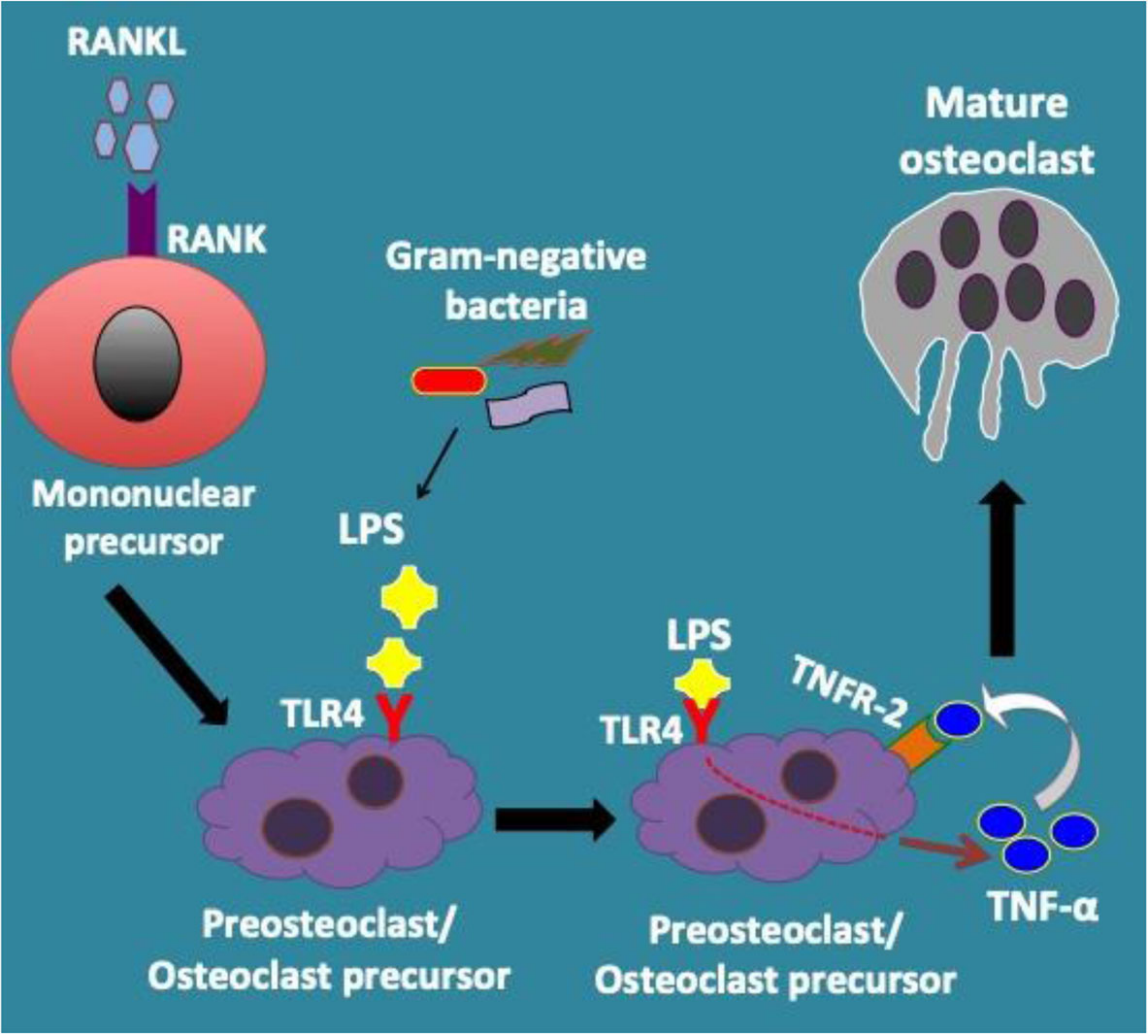
A schematic model is summarizing the possible role of LPS in osteoclastogenesis via TLR-4. Bacterial LPS induces osteoclast formation in RANKL-primed cells (pre-osteoclasts) via TLR4 signaling. LPS/TLR4 activation results in the secretion of TNF-α, which directly stimulates osteoclastogenic signals in pre-osteoclasts in a paracrine/autocrine manner via TNFR-2. LPS, lipopolysaccharide; RANKL, receptor activator of nuclear factor kappa-B ligand; TNF-α, tumor necrosis factor α; TLR4, Toll-like receptor 4; TNFR-2, tumor necrosis factor receptor-2

In conclusion, we illustrated the role of bacterial LPS in osteoclast differentiation (Fig. 6). The effect of LPS on osteoclastogenesis was primarily measured by TRAP staining. RAW cells failed to differentiate into TRAP-positive multinucleated cells when exposed to LPS alone without RANKL. However, LPS treatment of RANKL-primed cells significantly enhanced osteoclastogenesis. LPS/TLR4-induced TNF-α expression regulated osteoclast differentiation in RANKL-primed cells in an autocrine/paracrine manner. TNFR-2 seems to mediate LPS/TNF-α-mediated osteoclastogenesis. The RANKL/RANK/OPG axis was not activated during LPS-induced osteoclastogenesis.

Moreover, age- and post–menopausal–related osteoporosis are associated with chronic inflammation and immune system remodeling. Inflammatory mediators (e.g., TNF-α) are critical elements of pathological conditions observed in periodontitis, age-related osteoporosis, and rheumatoid arthritis. Our present study provides evidence for the molecular consequences of the regulatory mechanism (s) mediated by LPS-TNF-α and its receptors. However, additional studies are required to determine the underlying molecular mechanisms involved in osteoclastogenesis in response to LPS. Our results highlight a potent osteoclastogenesis process that occurs during bacteria-mediated osteolytic infections. Hence, targeting LPS/TNF-α-induced osteoclastogenesis could be a promising therapeutic strategy to inhibit inflammatory bone loss in osteolytic diseases such as periodontitis, rheumatoid arthritis, and osteoporosis.

## Methods

### Chemicals and reagents

RAW 264.7 cells were purchased from American Type Culture Collection (ATCC® TIB-71™). PG-LPS was purchased from InvivoGen (San Diego, CA, USA), dissolved in endotoxin-free water, and stored at − 20 °C. The Mem-PER™ Plus Membrane Protein Extraction Kit was purchased from Thermo Fisher Scientific (Cat. #89842; Waltham, MA, USA). Neutralizing antibodies against mouse TNF-α (AF410), recombinant mouse OPG (459MO), and mouse TNF-alpha Quantikine ELISA kit (MTA00B) were purchased from R&D Systems (Minneapolis, MN). The following antibodies were purchased from the company indicated in parentheses: RANK and TLR4 (SC-374360, SC-293072; Santa Cruz Biotechnology; Santa Cruz, CA), TNFR-1, and TNFR-2 (Abcam; Cambridge, United Kingdom). GAPDH antibody was purchased from Sigma-Aldrich (St. Louis, MO), and HRP-conjugated (mouse or rabbit) secondary antibodies were purchased from Santa Cruz Biotechnology (Santa Cruz, CA). Reagents for protein estimation, SDS-PAGE, molecular weight markers for SDS-PAGE were purchased from Bio-Rad. Chemiluminescent substrate and TLR4 inhibitor TAK-242 (508336) were obtained from Thermo Fisher Scientific (Waltham, MA).

### Preparation of osteoclast precursors from RAW 264.7 macrophage-like cell line

Osteoclasts were generated from RAW 264.7 cells, as described [59]. Mature multinucleated osteoclasts were observed from day 3 onwards. We used purified recombinant GST fused RANKL to differentiate osteoclasts, and the purification was done as described previously [60].

### Induction of osteoclast differentiation with RANKL or LPS and inhibition with OPG in the presence of RANKL or LPS

To determine the effect of OPG on RANKL- and LPS-induced osteoclastogenesis, RAW cells were treated with OPG at different stages. For RANKL-induced cultures, the treatment conditions were denoted as *control, late, and early + late* (illustrated in Additional file 2, Figure S2A). The treatment strategy was as follows:

In the *control* treatment, RAW cells were treated with RANKL (60 ng/mL of media) for 72 h. RANKL was added three times to the RAW cell culture at 24 h intervals (0 h, 24 h, and 48 h). Incubation was continued for 72 h, and osteoclasts were observed ~ 72 h after treatment with RANKL.

In the *late* treatment, RANKL and OPG were added to the RAW cell culture at 0 h, and incubation was continued 24 h. After 24 h, cells were washed with cold phosphate-buffered saline (PBS), and a fresh medium with RANKL was added at 24 h and 48 h intervals with no OPG. Incubation was continued for 72 h. OPG was added at a 2:1 ratio with RANKL (OPG 120 ng/mL; RANKL 60 ng/mL of media).

In the *early + late* treatment strategy*,* both RANKL (60 ng/mL of media) and OPG (120 ng/mL of media) were added at 0 h, 24 h, and 48 h. RANKL and OPG were present in the culture for 72 h.

In cultures treated with LPS, we used two conditions (illustrated in Fig. 2d). For the LPS control condition, RANKL (60 ng/mL of media) was added to RAW cells for the first 24 h. After 24 h, cells were washed with cold PBS, and the medium containing LPS (5 μg/mL of media) alone was added to the cultures. For the group treated with LPS and OPG, RAW cells were first treated with RANKL (60 ng/mL of media) for 24 h. After 24 h, cells were washed with cold PBS, and a medium containing LPS (5 μg/mL of media) + OPG (120 ng/mL medium) was added at 24 h and 48 h intervals. The incubation was continued for 72 h, as previously described [61]. OPG was added at the same concentration used for RANKL cultures (120 ng/mL of media).

### Inhibition of TRL4 with the inhibitor TAK-242

RAW cells were treated with TAK-242 (a selective TLR4 inhibitor) to analyze TLR4 signaling. The following groups were established (Table 1):

1. RANKL
2. RANKL+ TAK-242
3. LPS
4. LPS + TAK-242

**Table 1:** Treatment strategies for RAW cells in TLR4 inhibition experiments

Treatment Group	Treatment at 0 h	Treatment at 24 h	Treatment at 48 h
***RANKL***	RANKL (60 ng/mL)	RANKL (60 ng/mL)	RANKL (60 ng/mL)
***RANKL + TAK-242***	RANKL (60 ng/mL)	RANKL (60 ng/mL) + TAK-242 (5 μM/mL)	RANKL (60 ng/mL) + TAK-242 (5 μM/mL)
***LPS***	RANKL (60 ng/mL)	LPS (5 μg/mL)	LPS (5 μg/mL)
***LPS + TAK-242***	RANKL (60 ng/mL)	LPS (5 μg/mL) + TAK-242 (5 μM/mL)	LPS (5 μg/mL) + TAK-242 (5 μM/mL)

For treatment with TAK-242 in the presence of RANKL, RAW cells were treated with RANKL (60 ng/mL of media) at 0 h, and the incubation was continued for 24 h. After 24 h, cells were washed three times with cold PBS, and the medium was replaced with a fresh medium containing RANKL and TAK-242 (5 μM/mL) for another 24 h. This step was repeated at 48 h, and incubation was continued for 72 h. For controls in this experiment, cells treated with RANKL alone for 72 h were used (Table 1).

The LPS control group is described in the table below. For treatment with TAK-242 in the presence of LPS, RAW cells were treated with RANKL (60 ng/mL of media) at 0 h, and incubation was continued for 24 h. After 24 h, cells were washed three times with cold PBS, and the medium was replaced with fresh medium containing LPS (5 μg/mL) and TAK-242 (5 μM/mL) for another 24 h. This step was repeated at 48 h, and incubation was continued for 72 h (Table 1). After 72 h of incubation, cells were fixed and subjected to TRAP staining as described previously [62].

### Tartrate-resistant acid phosphatase (TRAP)-staining

TRAP staining was done for the most part, as described previously [62]. We used Leukocyte Acid Phosphatase Kit (Sigma; 387-A) and followed the manufacturer’s instructions. Stained cells were photographed with phase-contrast microscopy, and images were processed in Adobe Photoshop (Adobe Systems Inc.)

### Preparation of membrane lysates

RAW cells treated as above were used for the preparation of membrane fraction. Membrane-fraction was made using the Mem-PER™ Plus Membrane Protein Extraction Kit (Thermo Fisher Scientific, Cat. #89842). The procedure was performed according to instructions provided by the manufacturer. The protein concentration was determined using the Bradford assay.

### Immunoblotting analysis

Immunoblotting was performed as previously described [61, 62]. Briefly, an equal amount of lysate protein was separated by SDS-PAGE (10% gel) and transferred to polyvinylidene fluoride (PVDF) microporous membranes. Membranes were blocked and incubated with the primary and secondary antibody of interest in PBS-T at dilutions recommended by the manufacturer at 4 °C overnight. Membranes were washed three times with PBS-T for 5–10 min; protein bands were visualized by chemiluminescence using an ECL kit [63, 64]. The band densities were quantified and analyzed with UN-SCAN-IT software (Silk Science Inc., Orem, UT, USA). The final average pixel of the protein band of TNFR-1 and TNFR-2 was calculated by normalizing to the loading control protein GAPDH. The impact of various treatments was expressed either as percent surface level or fold change relative to the control lane.

### TNF-α measurement by ELISA

Briefly, RAW 264.7 cells were cultured in 6-well plates. After 24 h, cells were stimulated with RANKL and M-CSF for another 24 h. Cells were treated with RANKL, LPS (5 μg/mL), or LPS (5 μg/mL) + TAK-242 (5 μM/mL) for 48 h. The RANKL-treated cells were differentiated into osteoclasts and were considered as positive controls. The basal level of TNF-α was determined by measuring the untreated RAW 264.7 cells (−). After 48 h, supernatants were collected, and TNF-α levels were quantified with the mouse TNF-alpha Quantikine ELISA kit (MTA00B; R&D Systems; Minneapolis, MN) as per instructions provided by the manufacturer [65].

### Immunostaining

RANKL- and LPS-induced osteoclasts cultured on glass coverslips were immunostained with antibodies against RANK in 1:300 dilution (SC-374360; Santa Cruz Biotechnology; Santa Cruz, CA). After staining and mounting as previously described [66], osteoclasts were viewed using Cytation 5 cell imaging with the appropriate channel. Images were processed using Adobe Photoshop (Adobe Systems, Inc., Mountain View, CA).

### Statistical analysis

Statistical analyses were performed using Prism 8 software (GraphPad Inc. San Diego, CA). The statistical significance was determined using one-way ANOVA or Student’s t-test as applicable, and *p* values were considered significant when *p* < 0.05. Results are presented as mean ± standard deviation (SD).

## Supplementary Information

**Additional file 1: Figure S1.** Analysis of the potential osteoclastogenic effect of the LPS. (A) The diagrammatic sketch demonstrates the treatment strategy of RAW cells with RANKL and LPS. (B) And (C) representative images of TRAP stained osteoclasts (B) and RAW cells (C) in response to the treatment strategy shown in panel A. Images of different doses of LPS are shown in (C).

**Additional file 2: Figure S2.** Analysis of the effect of OPG treatment on RANKL-stimulated osteoclastogenesis. (A) Identification of the time-dependent effect of OPG on RANKL-induced osteoclast differentiation. The diagrammatic sketch demonstrates the treatment strategy of RAW cells with RANKL and OPG (120 ng/ml). (B) Representative images of TRAP stained osteoclasts in response to the treatment strategy shown in panel A. (C) The number of TRAP +ve multinucleated osteoclasts were counted in all treatment groups. Statistical analysis was performed to compare the number in the late and early+late treatment groups to the control group (RANKL). One-way ANOVA was applied, and the values were expressed as mean ± SD. *P < 0.05 vs. the control group (C).

**Additional file 3: Figure S3.** Immunoblotting analysis of the effect of OPG treatment on RANK expression. (A) An equal amount of membrane lysate proteins were used for immunoblotting analyses with antibodies to RANK (~90 kDa) and GAPDH (loading control; ~37 kDa). Protein levels were quantified by densitometry, corrected for the sample load based on GAPDH expression, and expressed as fold-decrease relative to the control lanes (RANKL and LPS). The results represent one of three experiments performed. (B) Uncropped raw data for the immunoblotting analyses shown in panel A are provided. Red rectangle indicates the proteins that are shown in panel A. The other lanes that were not marked by a rectangle in each autoradiogram represent different treatments which are not pertinent to the present studies.

**Additional file 4: Figure S4.** Effect of anti-TNF-α on the differentiation of osteoclasts. RAW cells subjected to differentiation in the presence of RANKL (A) and TNF-α (C) were treated with a TNF-α antibody for three days (B and D). Differentiation was blocked in cells treated with TNF-α antibody in TNF-α –treated cells (panel D) and not in RANKL treated cells (panel B). This result suggests that anti- TNF-α had no effect on the RANKL-RANK pathway involved in osteoclast differentiation. Representative phase-contrast microscopy images of TRAP-stained osteoclasts are shown. Cells were photographed under a 20X objective.

**Additional file 5: Figure S5.** Analysis of the RANK expression in LPS- and RANKL - mediated osteoclastogenesis. Uncropped raw data for the immunoblotting analyses shown in Fig. 2a are provided.

**Additional file 6: Figure S6.** Analysis of the TLR 4 level in untreated (RAW cells) treated cells with RANKL and LPS. Uncropped raw data for the immunoblotting analyses shown in Fig. 3a are provided.

**Additional file 7: Figure S7. **Immunoblotting analysis of membrane levels of TNFR-1 (panel A) and TNFR-2 in response to RANKL (R) and LPS-treatment. Uncropped raw data for the immunoblotting analyses shown in Fig. 5A for the membrane (surface) levels of TNFR-1 (A) and TNFR-2 (B) are provided. White rectangle in A indicates the TNFR1 protein band (~55kDa) and red rectangle in B panel 1) indicate the TNFR-2 (~68kDa) protein band of interest. Four blots for TNFR-1 (A) and three blots for TNFR-2 (B) are shown. TNFR-1 and TNFR-2 bands were scanned in Un-Scan-IT software and provided as percent surface levels in (Fig. 5C and D) in the manuscript. (C) Immunoblotting analysis of membrane levels of TNFR-1 and TNFR-2 in cells treated with LPS and LPS/anti-TNF-α. Uncropped raw data (Two autoradiogram for each experiment) of the immunoblotting analyses shown in Fig. 5B are provided. Blots were scanned and fold change in the levels of TNFR-1 and TNFR-2 are provided in (Fig. 5C-H) in the manuscript. The red rectangle in panel 3 (top) indicate the TNFR-2 (~68kDa) protein band. TNFR1 and TNFR-2 bands were scanned and provided as fold change in the surface levels in (Fig. 5 E-H) in the manuscript.

## Data Availability

The data sets used and/or analyzed during the current study are available from the corresponding author on reasonable request.

## Abbreviations

ELISA: Enzyme-linked immunosorbent assay
RANKL: Receptor activator of nuclear factor kappa-B ligand
M-CSF: Macrophage colony-stimulating factor
OPG: Osteoprotegerin
TLR4: Toll-like receptor 4
LPS: Lipopolysaccharide
TNF-α: Tumor necrosis factor α
TNFRs: Tumor necrosis factor receptors

## References

[1] Tella SH, Gallagher JC (2014). Biological agents in the management of osteoporosis. Eur J Clin Pharmacol.

[2] Hajishengallis G (2015). Periodontitis: from microbial immune subversion to systemic inflammation. Nat Rev Immunol.

[3] Azuma Y, Kaji K, Katogi R, Takeshita S, Kudo A (2000). Tumor necrosis factor-alpha induces differentiation of and bone resorption by osteoclasts. J Biol Chem.

[4] Yamaguchi R, Yoshimura A, Yoshioka H, Kaneko T, Hara Y (2009). Ability of supragingival plaque to induce toll-like receptor 4-mediated stimulation is associated with cytokine production by peripheral blood mononuclear cells. J Periodontol.

[5] Watanabe K, Iizuka T, Adeleke A, Pham L, Shlimon AE, Yasin M (2011). Involvement of toll-like receptor 4 in alveolar bone loss and glucose homeostasis in experimental periodontitis. J Periodontal Res.

[6] Vaananen K (2005). Mechanism of osteoclast mediated bone resorption--rationale for the design of new therapeutics. Adv Drug Deliv Rev.

[7] Abercrombie M (1980). The crawling movement of metazoan cells. Proc Natl Acad Sci U S A.

[8] Boyle WJ, Simonet WS, Lacey DL (2003). Osteoclast differentiation and activation. Nature.

[9] Asagiri M, Takayanagi H (2007). The molecular understanding of osteoclast differentiation. Bone.

[10] Kearns AE, Khosla S, Kostenuik PJ (2008). Receptor activator of nuclear factor kappaB ligand and osteoprotegerin regulation of bone remodeling in health and disease. Endocr Rev.

[11] Nagasawa T, Kiji M, Yashiro R, Hormdee D, Lu H, Kunze M (2007). Roles of receptor activator of nuclear factor-kappaB ligand (RANKL) and osteoprotegerin in periodontal health and disease. Periodontol.

[12] Kawai T, Matsuyama T, Hosokawa Y, Makihira S, Seki M, Karimbux NY (2006). B and T lymphocytes are the primary sources of RANKL in the bone resorptive lesion of periodontal disease. Am J Pathol.

[13] Algate K, Haynes DR, Bartold PM, Crotti TN, Cantley MD (2016). The effects of tumour necrosis factor-alpha on bone cells involved in periodontal alveolar bone loss; osteoclasts, osteoblasts and osteocytes. J Periodontal Res.

[14] Kato H, Taguchi Y, Tominaga K, Umeda M, Tanaka A (2014). Porphyromonas gingivalis LPS inhibits osteoblastic differentiation and promotes pro-inflammatory cytokine production in human periodontal ligament stem cells. Arch Oral Biol.

[15] Kawai T, Akira S (2010). The role of pattern-recognition receptors in innate immunity: update on toll-like receptors. Nat Immunol.

[16] Ogawa T, Yagi T (2010). Bioactive mechanism of Porphyromonas gingivalis lipid A. Periodontol.

[17] Hajishengallis G, Darveau RP, Curtis MA (2012). The keystone-pathogen hypothesis. Nat Rev Microbiol.

[18] Silva N, Abusleme L, Bravo D, Dutzan N, Garcia-Sesnich J, Vernal R (2015). Host response mechanisms in periodontal diseases. J Appl Oral Sci.

[19] Kawasaki T, Kawai T (2014). Toll-like receptor signaling pathways. Front Immunol.

[20] Cekici A, Kantarci A, Hasturk H, Van Dyke TE (2014). Inflammatory and immune pathways in the pathogenesis of periodontal disease. Periodontol.

[21] Estornes Y, Bertrand MJ (2015). IAPs, regulators of innate immunity and inflammation. Semin Cell Dev Biol.

[22] Fujihara M, Muroi M, Tanamoto K, Suzuki T, Azuma H, Ikeda H (2003). Molecular mechanisms of macrophage activation and deactivation by lipopolysaccharide: roles of the receptor complex. Pharmacol Ther.

[23] Wong M, Ziring D, Korin Y, Desai S, Kim S, Lin J (2008). TNFalpha blockade in human diseases: mechanisms and future directions. Clin Immunol.

[24] Idriss HT, Naismith JH (2000). TNF alpha and the TNF receptor superfamily: structure-function relationship(s). Microsc Res Tech.

[25] Xu Y, Chang L, Huang A, Liu X, Liu X, Zhou H (2017). Functional detection of TNF receptor family members by affinity-labeled ligands. Sci Rep.

[26] Zhang YH, Heulsmann A, Tondravi MM, Mukherjee A, Abu-Amer Y (2001). Tumor necrosis factor-alpha (TNF) stimulates RANKL-induced osteoclastogenesis via coupling of TNF type 1 receptor and RANK signaling pathways. J Biol Chem.

[27] Kobayashi K, Takahashi N, Jimi E, Udagawa N, Takami M, Kotake S (2000). Tumor necrosis factor alpha stimulates osteoclast differentiation by a mechanism independent of the ODF/RANKL-RANK interaction. J Exp Med.

[28] Lam J, Takeshita S, Barker JE, Kanagawa O, Ross FP, Teitelbaum SL (2000). TNF-alpha induces osteoclastogenesis by direct stimulation of macrophages exposed to permissive levels of RANK ligand. J Clin Invest.

[29] Kudo O, Fujikawa Y, Itonaga I, Sabokbar A, Torisu T, Athanasou NA (2002). Pro-inflammatory cytokine (TNFalpha/IL-1alpha) induction of human osteoclast formation. J Pathol.

[30] Fuller K, Murphy C, Kirstein B, Fox SW, Chambers TJ (2002). TNFalpha potently activates osteoclasts, through a direct action independent of and strongly synergistic with RANKL. Endocrinology.

[31] Abu-Amer Y, Ross FP, Edwards J, Teitelbaum SL (1997). Lipopolysaccharide-stimulated osteoclastogenesis is mediated by tumor necrosis factor via its P55 receptor. J Clin Invest.

[32] Nakanishi-Matsui M, Yano S, Matsumoto N, Futai M (2012). Lipopolysaccharide induces multinuclear cell from RAW264.7 line with increased phagocytosis activity. Biochem Biophys Res Commun.

[33] Hou GQ, Guo C, Song GH, Fang N, Fan WJ, Chen XD (2013). Lipopolysaccharide (LPS) promotes osteoclast differentiation and activation by enhancing the MAPK pathway and COX-2 expression in RAW264.7 cells. Int J Mol Med.

[34] Chellaiah MA, Majumdar S, Aljohani H (2018). Peptidomimetic inhibitors of L-plastin reduce the resorptive activity of osteoclast but not the bone forming activity of osteoblasts in vitro. PLoS One.

[35] Hayman AR (2008). Tartrate-resistant acid phosphatase (TRAP) and the osteoclast/immune cell dichotomy. Autoimmunity.

[36] Gao A, Wang X, Yu H, Li N, Hou Y, Yu W (2016). Effect of Porphyromonas gingivalis lipopolysaccharide (Pg-LPS) on the expression of EphA2 in osteoblasts and osteoclasts. In Vitro Cell Dev Biol Anim.

[37] Nativel B, Couret D, Giraud P, Meilhac O, d’Hellencourt CL, Viranaicken W (2017). Porphyromonas gingivalis lipopolysaccharides act exclusively through TLR4 with a resilience between mouse and human. Sci Rep.

[38] Darveau RP, Arbabi S, Garcia I, Bainbridge B, Maier RV (2002). Porphyromonas gingivalis lipopolysaccharide is both agonist and antagonist for p38 mitogen-activated protein kinase activation. Infect Immun.

[39] Taubman MA, Valverde P, Han X, Kawai T (2005). Immune response: the key to bone resorption in periodontal disease. J Periodontol.

[40] Boyce BF, Xing L (2007). The RANKL/RANK/OPG pathway. Curr Osteoporos Rep.

[41] Ii M, Matsunaga N, Hazeki K, Nakamura K, Takashima K, Seya T (2006). A novel cyclohexene derivative, ethyl (6R)-6-[N-(2-Chloro-4-fluorophenyl)sulfamoyl] cyclohex-1-ene-1-carboxylate (TAK-242), selectively inhibits toll-like receptor 4-mediated cytokine production through suppression of intracellular signaling. Mol Pharmacol.

[42] Ye LL, Wei XS, Zhang M, Niu YR, Zhou Q (2018). The Significance of Tumor Necrosis Factor Receptor Type II in CD8(+) Regulatory T Cells and CD8(+) Effector T Cells. Front Immunol.

[43] Zaidi M (2007). Skeletal remodeling in health and disease 37. Nat Med.

[44] Socransky SS, Haffajee AD, Cugini MA, Smith C, Kent RL (1998). Microbial complexes in subgingival plaque5. J Clin Periodontol.

[45] Cheng X, Kinosaki M, Murali R, Greene MI (2003). The TNF receptor superfamily: role in immune inflammation and bone formation7. Immunol Res.

[46] Liu J, Wang S, Zhang P, Said-Al-Naief N, Michalek SM, Feng X (2009). Molecular mechanism of the bifunctional role of lipopolysaccharide in osteoclastogenesis 298. J Biol Chem.

[47] Zhang P, Liu J, Xu Q, Harber G, Feng X, Michalek SM (2011). TLR2-dependent modulation of osteoclastogenesis by Porphyromonas gingivalis through differential induction of NFATc1 and NF-kappaB 44. J Biol Chem.

[48] Seidler NW (2013). Compartmentation of GAPDH. Adv Exp Med Biol.

[49] Sirover MA (2012). Subcellular dynamics of multifunctional protein regulation: mechanisms of GAPDH intracellular translocation. J Cell Biochem.

[50] Terrasse R, Tacnet-Delorme P, Moriscot C, Perard J, Schoehn G, Vernet T (2012). Human and pneumococcal cell surface glyceraldehyde-3-phosphate dehydrogenase (GAPDH) proteins are both ligands of human C1q protein. J Biol Chem.

[51] AlQranei MS, Chellaiah MA (2020). Osteoclastogenesis in periodontal diseases: possible mediators and mechanisms. J Oral Biosci.

[52] Lee HK, Lee J, Tobias PS (2002). Two lipoproteins extracted from *Escherichia coli* K-12 LCD25 lipopolysaccharide are the major components responsible for Toll-like receptor 2-mediated signaling. J Immunol.

[53] Hirschfeld M, Ma Y, Weis JH, Vogel SN, Weis JJ (2000). Cutting edge: repurification of lipopolysaccharide eliminates signaling through both human and murine toll-like receptor 2. J Immunol.

[54] Matsunaga N, Tsuchimori N, Matsumoto T, Ii M (2011). TAK-242 (resatorvid), a small-molecule inhibitor of toll-like receptor (TLR) 4 signaling, binds selectively to TLR4 and interferes with interactions between TLR4 and its adaptor molecules. Mol Pharmacol.

[55] Takami M, Kim N, Rho J, Choi Y (2002). Stimulation by toll-like receptors inhibits osteoclast differentiation. J Immunol.

[56] Staab E, Thiele GM, Clarey D, Wyatt TA, Romberger DJ, Wells AD (2016). Toll-like receptor 4 signaling pathway mediates inhalant organic dust-induced bone loss. PLoS ONE.

[57] Itoh K, Udagawa N, Kobayashi K, Suda K, Li X, Takami M (2003). Lipopolysaccharide promotes the survival of osteoclasts via toll-like receptor 4, but cytokine production of osteoclasts in response to lipopolysaccharide is different from that of macrophages. J Immunol.

[58] Hussain MA, Saito H, Alles N, Shimokawa H, Aoki K, Ohya K (2008). Lipopolysaccharide-induced bone resorption is increased in TNF type 2 receptor-deficient mice in vivo. J Bone Miner Metab.

[59] Gupta A, Lee BS, Khadeer MA, Tang Z, Chellaiah M, Abu-Amer Y (2003). Leupaxin is a critical adaptor protein in the adhesion zone of the osteoclast. J Bone Miner Res.

[60] Ma T, Sadashivaiah K, Chellaiah MA (2010). Regulation of sealing ring formation by L-plastin and cortactin in osteoclasts. J Biol Chem.

[61] AlQranei MS, Aljohani H, Majumdar S, Senbanjo LT, Chellaiah MA (2020). C-phycocyanin attenuates RANKL-induced osteoclastogenesis and bone resorption in vitro through inhibiting ROS levels, NFATc1 and NF-kappaB activation. Sci Rep.

[62] Aljohani H, Senbanjo LT, Chellaiah MA (2019). Methylsulfonylmethane increases osteogenesis and regulates the mineralization of the matrix by transglutaminase 2 in SHED cells. PLoS ONE.

[63] Chellaiah M, Hruska KA (1996). Osteopontin stimulates gelsolin associated phosphoinositide levels and PtdIns 3-hydroxyl kinase. Mol Biol Cell.

[64] Chellaiah MA, Kizer N, Biswas R, Alvarez U, Strauss-Schoenberger J, Rifas L (2003). Osteopontin deficiency produces osteoclast dysfunction due to reduced CD44 surface expression. Mol Biol Cell.

[65] Perez-Aso M, Montesinos MC, Mediero A, Wilder T, Schafer PH, Cronstein B (2015). Apremilast, a novel phosphodiesterase 4 (PDE4) inhibitor, regulates inflammation through multiple cAMP downstream effectors. Arthritis Res Ther.

[66] Chellaiah MA, Biswas RS, Rittling SR, Denhardt DT, Hruska KA (2003). Rho-dependent rho kinase activation increases CD44 surface expression and bone resorption in osteoclasts. J Biol Chem.

