# Supplementary material for: Lipopolysaccharide- TLR-4 Axis regulates Osteoclastogenesis independent of RANKL/RANK signaling
Source: BMC Immunol. 2021 Mar 25;22:23. doi: 10.1186/s12865-021-00409-9 (PMC7995782; doi:10.1186/s12865-021-00409-9)
Supplement: Supplementary file 1 — Additional file 1: Figure S1. Analysis of the potential osteoclastogenic effect of the LPS. (A) The diagrammatic sketch demonstrates the treatment strategy of RAW cells with RANKL and LPS. (B) And (C) representative images of TRAP stained osteoclasts (B) and RAW cells (C) in response to the treatment strategy shown in panel A. Images of different doses of LPS are shown in (C). [file 12865_2021_409_MOESM1_ESM.docx]

**Additional File. 1**


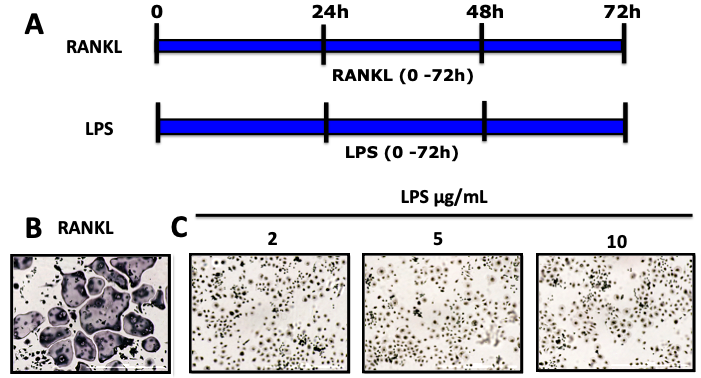


**Additional Figure S1: Analysis of the potential osteoclastogenic effect of the LPS.**

**(A)** The diagrammatic sketch demonstrates the treatment strategy of RAW cells with RANKL and LPS. **(B)** And **(C)** representative images of TRAP stained osteoclasts **(B)** and RAW cells **(C)** in response to the treatment strategy shown in panel **A.** Images of different doses of LPS are shown in **(C).**
