# Supplementary material for: Lipopolysaccharide- TLR-4 Axis regulates Osteoclastogenesis independent of RANKL/RANK signaling
Source: BMC Immunol. 2021 Mar 25;22:23. doi: 10.1186/s12865-021-00409-9 (PMC7995782; doi:10.1186/s12865-021-00409-9)
Supplement: Supplementary file 2 — Additional file 2: Figure S2. Analysis of the effect of OPG treatment on RANKL-stimulated osteoclastogenesis. (A) Identification of the time-dependent effect of OPG on RANKL-induced osteoclast differentiation. The diagrammatic sketch demonstrates the treatment strategy of RAW cells with RANKL and OPG (120 ng/ml). (B) Representative images of TRAP stained osteoclasts in response to the treatment strategy shown in panel A. (C) The number of TRAP +ve multinucleated osteoclasts were counted in all treatment groups. Statistical analysis was performed to compare the number in the late and early+late treatment groups to the control group (RANKL). One-way ANOVA was applied, and the values were expressed as mean ± SD. *P < 0.05 vs. the control group (C). [file 12865_2021_409_MOESM2_ESM.docx]

**Additional Figure S2: Analysis of the effect of OPG treatment on RANKL-stimulated osteoclastogenesis.**

**(A)** Identification of the time-dependent effect of OPG on RANKL-induced osteoclast differentiation. The diagrammatic sketch demonstrates the treatment strategy of RAW cells with RANKL and OPG (120 ng/ml). **(B)** Representative images of TRAP stained osteoclasts in response to the treatment strategy shown in panel **A. (C)** The number of TRAP +ve multinucleated osteoclasts were counted in all treatment groups. Statistical analysis was performed to compare the number in the *late* and *early+late* treatment groups to the control group (RANKL). One-way ANOVA was applied, and the values were expressed as mean ± SD. *P < 0.05 vs. the control group **(C).**


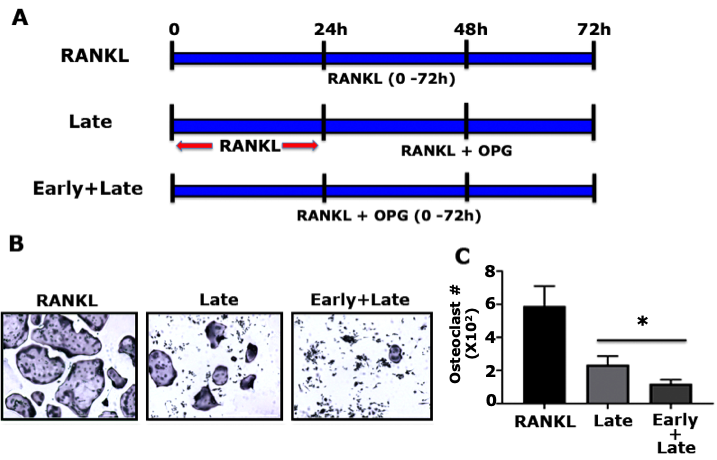


**Additional File. 2**
