# Supplementary material for: Lipopolysaccharide- TLR-4 Axis regulates Osteoclastogenesis independent of RANKL/RANK signaling
Source: BMC Immunol. 2021 Mar 25;22:23. doi: 10.1186/s12865-021-00409-9 (PMC7995782; doi:10.1186/s12865-021-00409-9)
Supplement: Supplementary file 3 — Additional file 3: Figure S3. Immunoblotting analysis of the effect of OPG treatment on RANK expression. (A) An equal amount of membrane lysate proteins were used for immunoblotting analyses with antibodies to RANK (~90 kDa) and GAPDH (loading control; ~37 kDa). Protein levels were quantified by densitometry, corrected for the sample load based on GAPDH expression, and expressed as fold-decrease relative to the control lanes (RANKL and LPS). The results represent one of three experiments performed. (B) Uncropped raw data for the immunoblotting analyses shown in panel A are provided. Red rectangle indicates the proteins that are shown in panel A. The other lanes that were not marked by a rectangle in each autoradiogram represent different treatments which are not pertinent to the present studies. [file 12865_2021_409_MOESM3_ESM.docx]

**Additional File. 3**


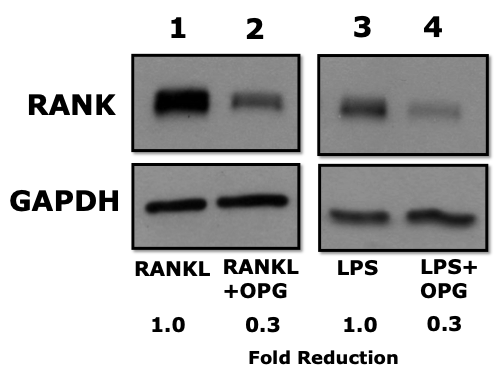

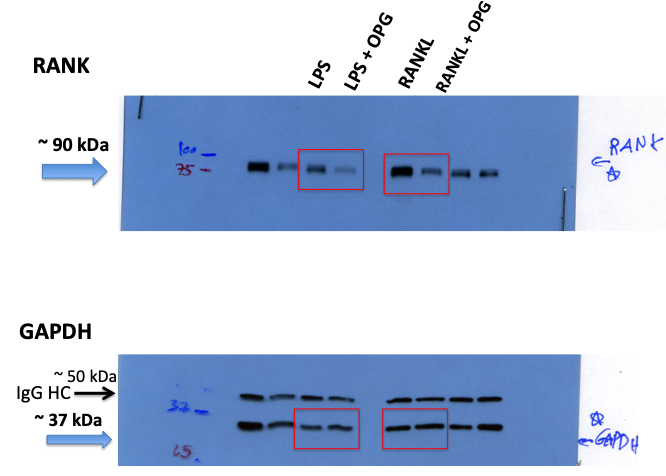


**Additional Figure S3: Immunoblotting analysis of the effect of OPG treatment on RANK expression.**

**(A)** An equal amount of membrane lysate proteins were used for immunoblotting analyses with antibodies to RANK (**~**90 kDa) and GAPDH (loading control; **~**37 kDa). Protein levels were quantified by densitometry, corrected for the sample load based on GAPDH expression, and expressed as fold-decrease relative to the control lanes (RANKL and LPS). The results represent one of three experiments performed.

**(B)** Uncropped raw data for the immunoblotting analyses shown in panel **A** are provided. Red rectangle indicates the proteins that are shown in panel **A**. The other lanes that were not marked by a rectangle in each autoradiogram represent different treatments which are not pertinent to the present studies.
