# Supplementary material for: Lipopolysaccharide- TLR-4 Axis regulates Osteoclastogenesis independent of RANKL/RANK signaling
Source: BMC Immunol. 2021 Mar 25;22:23. doi: 10.1186/s12865-021-00409-9 (PMC7995782; doi:10.1186/s12865-021-00409-9)
Supplement: Supplementary file 4 — Additional file 4: Figure S4. Effect of anti-TNF-α on the differentiation of osteoclasts. RAW cells subjected to differentiation in the presence of RANKL (A) and TNF-α (C) were treated with a TNF-α antibody for three days (B and D). Differentiation was blocked in cells treated with TNF-α antibody in TNF-α –treated cells (panel D) and not in RANKL treated cells (panel B). This result suggests that anti- TNF-α had no effect on the RANKL-RANK pathway involved in osteoclast differentiation. Representative phase-contrast microscopy images of TRAP-stained osteoclasts are shown. Cells were photographed under a 20X objective. [file 12865_2021_409_MOESM4_ESM.docx]

**Additional File. 4**


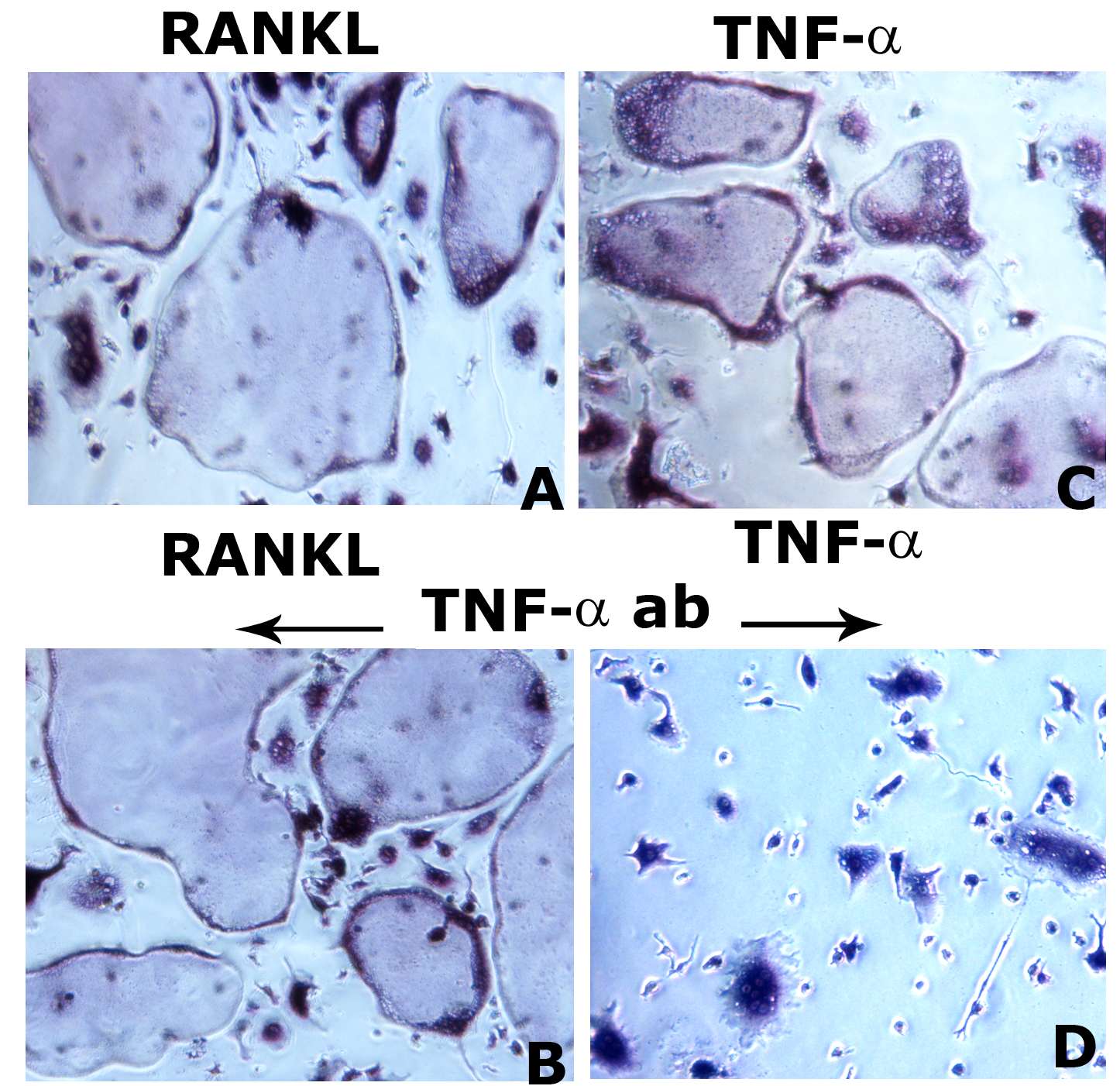


**Additional Figure S4: Effect of anti-TNF-α on the differentiation of osteoclasts**.

RAW cells subjected to differentiation in the presence of RANKL (A) and TNF-α (C) were treated with a TNF-α antibody for three days (B and D). Differentiation was blocked in cells treated with TNF-α antibody in TNF-α –treated cells (panel D) and not in RANKL treated cells (panel B). This result suggests that anti- TNF-α had no effect on the RANKL-RANK pathway involved in osteoclast differentiation. Representative phase-contrast microscopy images of TRAP-stained osteoclasts are shown. Cells were photographed under a 20X objective.
