# Supplementary material for: Lipopolysaccharide- TLR-4 Axis regulates Osteoclastogenesis independent of RANKL/RANK signaling
Source: BMC Immunol. 2021 Mar 25;22:23. doi: 10.1186/s12865-021-00409-9 (PMC7995782; doi:10.1186/s12865-021-00409-9)
Supplement: Supplementary file 5 — Additional file 5: Figure S5. Analysis of the RANK expression in LPS- and RANKL - mediated osteoclastogenesis. Uncropped raw data for the immunoblotting analyses shown in Fig. 2a are provided. [file 12865_2021_409_MOESM5_ESM.docx]

**Additional Figure S5: Analysis of the RANK expression in LPS- and RANKL - mediated osteoclastogenesis.**

Uncropped raw data for the immunoblotting analyses shown in Figure 2A are provided.


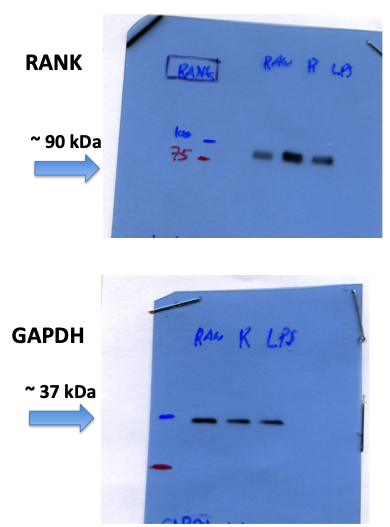


**Additional File. 5**
