# Supplementary material for: Lipopolysaccharide- TLR-4 Axis regulates Osteoclastogenesis independent of RANKL/RANK signaling
Source: BMC Immunol. 2021 Mar 25;22:23. doi: 10.1186/s12865-021-00409-9 (PMC7995782; doi:10.1186/s12865-021-00409-9)
Supplement: Supplementary file 6 — Additional file 6: Figure S6. Analysis of the TLR 4 level in untreated (RAW cells) treated cells with RANKL and LPS. Uncropped raw data for the immunoblotting analyses shown in Fig. 3a are provided. [file 12865_2021_409_MOESM6_ESM.docx]

**Additional Figure S6: Analysis of the TLR 4 level in untreated (RAW cells) treated cells with RANKL and LPS**

Uncropped raw data for the immunoblotting analyses shown in Figure 3A are provided.


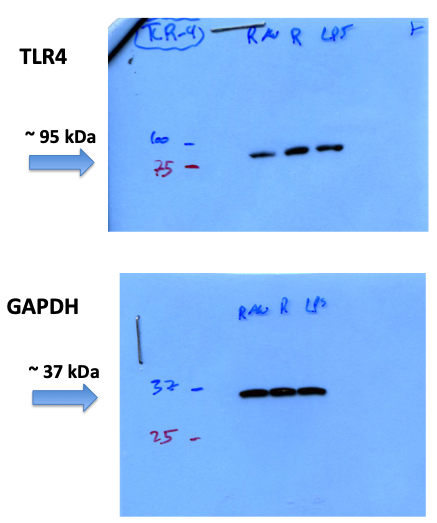


**Additional File. 6**
