# Supplementary material for: Lipopolysaccharide- TLR-4 Axis regulates Osteoclastogenesis independent of RANKL/RANK signaling
Source: BMC Immunol. 2021 Mar 25;22:23. doi: 10.1186/s12865-021-00409-9 (PMC7995782; doi:10.1186/s12865-021-00409-9)
Supplement: Supplementary file 7 — Additional file 7: Figure S7. Immunoblotting analysis of membrane levels of TNFR-1 (panel A) and TNFR-2 in response to RANKL (R) and LPS-treatment. Uncropped raw data for the immunoblotting analyses shown in Fig. 5A for the membrane (surface) levels of TNFR-1 (A) and TNFR-2 (B) are provided. White rectangle in A indicates the TNFR1 protein band (~55kDa) and red rectangle in B panel 1) indicate the TNFR-2 (~68kDa) protein band of interest. Four blots for TNFR-1 (A) and three blots for TNFR-2 (B) are shown. TNFR-1 and TNFR-2 bands were scanned in Un-Scan-IT software and provided as percent surface levels in (Fig. 5C and D) in the manuscript. (C) Immunoblotting analysis of membrane levels of TNFR-1 and TNFR-2 in cells treated with LPS and LPS/anti-TNF-α. Uncropped raw data (Two autoradiogram for each experiment) of the immunoblotting analyses shown in Fig. 5B are provided. Blots were scanned and fold change in the levels of TNFR-1 and TNFR-2 are provided in (Fig. 5C-H) in the manuscript. The red rectangle in panel 3 (top) indicate the TNFR-2 (~68kDa) protein band. TNFR1 and TNFR-2 bands were scanned and provided as fold change in the surface levels in (Fig. 5 E-H) in the manuscript. [file 12865_2021_409_MOESM7_ESM.docx]

**Additional File. 7**


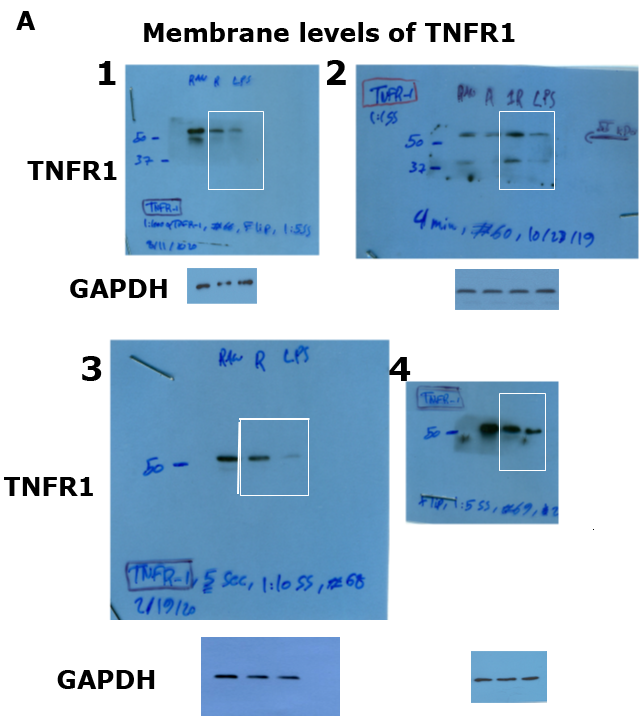


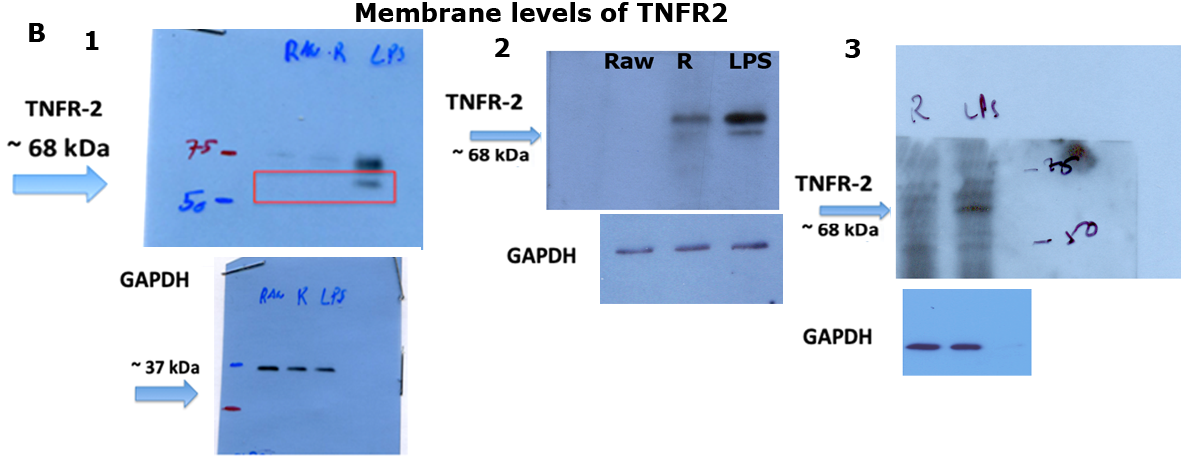


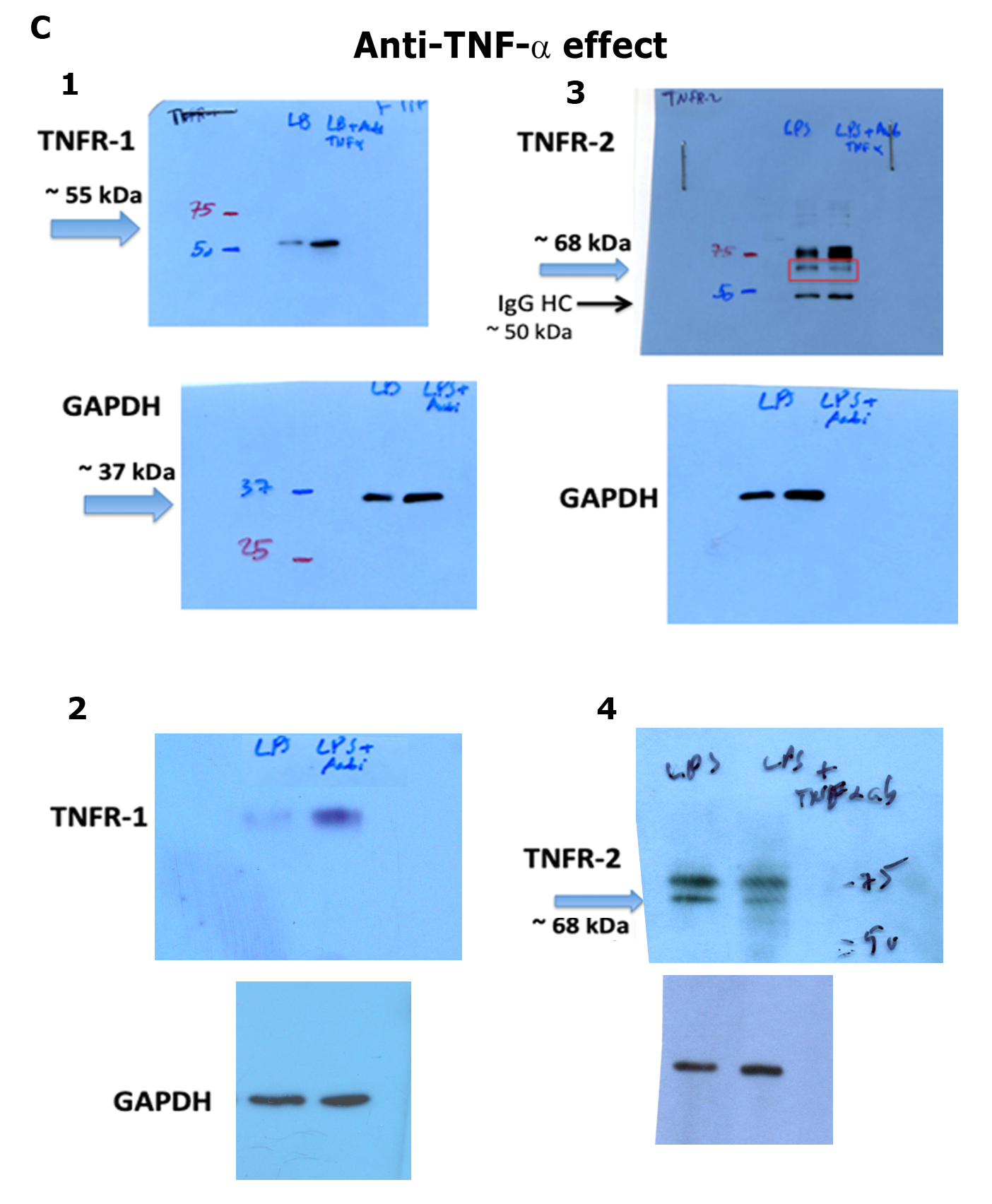


**Additional Figure S7C: Immunoblotting analysis of membrane levels of TNFR-1 and TNFR-2 in cells treated with LPS and LPS/anti-TNF-α**

Uncropped raw data (Two autoradiogram for each experiment) of the immunoblotting analyses shown in Figure 5B are provided. Blots were scanned and fold change in the levels of TNFR-1 and TNFR-2 are provided in Figure 5 (C-H) in the manuscript. The red rectangle in panel 3 (top) indicate the TNFR-2 (~68kDa) protein band. TNFR1 and TNFR-2 bands were scanned and provided as fold change in the surface levels in Figure 5 (E-H) in the manuscript.

**Additional Figure S7(A and B): Immunoblotting analysis of membrane levels of TNFR-1 (panel A) and TNFR-2 in response to RANKL (R) and LPS-treatment.**

Uncropped raw data for the immunoblotting analyses shown in Figure 5A for the membrane (surface) levels of TNFR-1 (A) and TNFR-2 (B) are provided. White rectangle in A indicates the TNFR1 protein band (~55kDa) and red rectangle in B panel 1) indicate the TNFR-2 (~68kDa) protein band of interest. Four blots for TNFR-1 (A) and three blots for TNFR-2 (B) are shown. TNFR-1 and TNFR-2 bands were scanned in Un-Scan-IT software and provided as percent surface levels in Figure 5 (C and D) in the manuscript.
